# The Efficacy of Trastuzumab in Animal Models of Breast Cancer: A Systematic Review and Meta-Analysis

**DOI:** 10.1371/journal.pone.0158240

**Published:** 2016-07-27

**Authors:** Jiarong Chen, Canhong Yang, Bin Guo, Emily S. Sena, Malcolm R. Macleod, Yawei Yuan, Theodore C. Hirst

**Affiliations:** 1 Department of Oncology, Jiangmen Central Hospital, Affiliated Jiangmen Hospital of Sun Yat-Sen University, Jiangmen, Guangdong 529030, P. R. China; 2 Department of Radiation Oncology, Nanfang Hospital, Southern Medical University, 1838 Guangzhou DaDao Bei, Guangzhou, Guangdong 510515, P. R. China; 3 Department of Neurology, the Third Affiliated Hospital of Southern Medical University, Guangzhou 510630, P. R. China; 4 Department of Hepatobiliary Surgery, Zhujiang Hospital, Southern Medical University, Guangzhou 510282, P. R. China; 5 Centre for Clinical Brain Sciences, Chancellors Building, University of Edinburgh, 49 Little France Crescent, Edinburgh EH16 4SB, United Kingdom; Fondazione IRCCS Istituto Nazionale dei Tumori, ITALY

## Abstract

**Background:**

Breast cancer is the most frequent cancers and is the second leading cause of cancer death among women. Trastuzumab is an effective treatment, the first monoclonal antibody directed against the human epidermal growth factor receptor 2 (HER2). To inform the development of other effective treatments we report summary estimates of efficacy of trastuzumab on survival and tumour volume in animal models of breast cancer.

**Methods:**

We searched PubMed and EMBASE systematically to identify publications testing trastuzumab in animal models of breast cancer. Data describing tumour volume, median survival and animal features were extracted and we assessed quality using a 12-item checklist. We analysed the impact of study design and quality and evidence for publication bias.

**Results:**

We included data from 83 studies reporting 169 experiments using 2076 mice. Trastuzumab treatment caused a substantial reduction in tumour growth, with tumours in treated animals growing to 32.6% of the volume of tumours in control animals (95%CI 27.8%-38.2%). Median survival was prolonged by a factor of 1.45 (1.30–1.62). Many study design and quality features accounted for between-study heterogeneity and we found evidence suggesting publication bias.

**Conclusion:**

We have found trastuzumab to be effective in animal breast cancer models across a range of experimental circumstances. However the presence of publication bias and a low prevalence of measures to reduce bias provide a focus for future improvements in preclinical breast cancer research.

## Introduction

Breast cancer is the second most common cancer in the world. With an estimated 1.67 million new cancer cases diagnosed in 2012, breast cancer is the most frequent cancer and second leading cause of cancer death among women [1]. Treatment options including surgery, radiation therapy, and systemic therapy have improved the survival and quality of life of breast cancer patients over the past 30 years [2]. Targeted therapies, which selectively focus on specific molecular targets on tumour cells, provide potential to improve survival and quality of life and to reduce drug side effects [3, 4].

The human epidermal growth factor receptor-2 (HER2), overexpressed in between 15% and 20% of breast cancers [5], is one of the most common molecular targets for targeted therapies [2]. Described as a proto-oncogene, HER2 expression in human cancer was first observed during screening of DNA samples derived from breast cancers [6]. HER2 gene amplification significantly predicts both reduced overall survival and shorter time to relapse in patients with breast cancer. Higher expression of HER2 is associated with a more aggressive disease course leading to worse clinical outcomes [6] as well as adverse prognostic features including advanced pathologic stage [7] and number of metastatic axillary lymph nodes [8]. As a predictor of risk of occurrence and response to therapy HER2 testing is recommended in pathology examination for all newly diagnosed invasive breast cancer and first recurrences of breast cancer [5, 9] as part of to inform staging and choice of therapy.

Trastuzumab, also known as Herceptin, is the first monoclonal antibody directed against HER2. Following studies in tumour xenografts showing that murine monoclonal antibody (MAb) 4D5 inhibits the proliferation of human breast cancer cells overexpressing the HER2 receptor [10], trastuzumab was created in 1990 by humanizing the 4D5 mouse antibody to overcome immunogenicity issues [11]. Subsequent first-generation clinical trials demonstrated prolonged survival and time to progression in patients with HER2-positive metastatic breast cancer [12]. As such, trastuzumab has been widely promoted and recommended for HER2-positive breast cancer in combination with chemotherapies such as cisplatin, doxorubicin or paclitaxel [13–15]. Several studies reported improved disease-free and overall survival following 1 year of trastuzumab administered concomitantly or sequentially as an adjuvant to chemotherapy [16]. Furthermore, trastuzumab shows some efficacy when administrated as a single agent in first-line treatment [17].

Trastuzumab has been used clinically for breast cancer since the mid-1990s and experience in humans has been summarised in systematic reviews and meta-analyses [18, 19]. However, efficacy in animal models has not been well characterised and the extent to which animal studies reliably informed early clinical trials is unclear. Animal experiments improve our understanding of disease mechanisms and they are invaluable for clinical research, ultimately informing decisions on when to proceed to clinical trial. Systematic review and meta-analysis of such studies may aid in the selection of the most promising treatment strategies for future clinical trials, as well as in the identification of potential sources of bias arising from limitations in study design [20]. In the context of treatments known to be effective in humans, a deeper understanding of these strengths and weaknesses of *in vivo* studies may inform the development of new therapies. The Collaborative Approach to Meta-Analysis and Review of Animal Data in Experimental Studies (CAMARADES) group have shown—for a range of experimental neurological diseases including glioma, stroke and multiple sclerosis—that reporting of measures to reduce risk of bias (such as randomisation and blinding) is poor and that publication bias is frequently present. These factors lead to a significant overstatement of perceived treatment efficacy [21–23].

Therefore, we aimed to undertake a systematic review of trastuzumab in animal models of breast cancer, to summarise the effect of trastuzumab on survival and tumour volume. We also sought to describe the impact on observed efficacy of study quality features such as randomization and blinding and experimental design features such as drug dose, timing of treatment, species, study quality, so that we might ascertain the value of such studies in predicting clinical efficacy and suggest how future animal research might be streamlined to predict clinical efficacy more reliably.

## Materials and Methods

The study was conducted in accordance with a protocol, first published online in December 2014 at http://www.dcn.ed.ac.uk/camarades/research.html#protocols.

### Search strategy

We searched PubMed and EMBASE on 4 July 2013, using the terms relating to (breast tumor OR breast cancer) AND (trastuzumab OR Herceptin) (S1 Table). On 18 September 2014, we added three further terms to expand the search with previous names of trastuzumab: (breast tumor OR breast cancer) AND ("rhuMAb HER2" OR "Anti-p185HER2 Monoclonal Antibody" OR "muMAb 4D5”). For EMBASE the search was limited to animals, and for PubMed we used a validated animal search filter [24]. Studies in all languages were accepted.

### Study selection

Two investigators independently screened all titles and abstracts to identify studies meeting the inclusion criteria. The full text of all these potentially eligible studies were retrieved and independently assessed for eligibility by two investigators (Canhong Yang and Bin Guo), disagreements were discussed with a third investigator (Jiarong Chen) after full text reading. We included controlled studies that reported the efficacy of trastuzumab monotherapy in laboratory animals with induced breast cancer. We included studies reporting outcome as either median survival or tumour volume in treatment and control groups. Publications had to state the number of animals per group, and we excluded Reviews, Books, Letters, Clinical trials, Case reports, or Editorials.

### Data extraction

We extracted data to the CAMARADES data manager (Microsoft Access) for assessment of study quality and evidence synthesis. Extracted information included: article information (title, author, journal, publication year); agent for treatment and control group; trastuzumab treatment (dose, total dose and frequency, route of administration, delay to treatment); tumour volume data for treatment and control group (original tumour size included, measurement method and frequency, measurement day after treatment, measurement day after tumour implantation), and whether the study reported the blinded assessment of outcome. We recorded data from the last time point for tumour volume measurement for treatment and control group; median survival data for treatment and control group; experimental animals (number of animals for every group, species and strain, age, sex and original weight), breast cancer model (breast cancer cell type, HER2 expression level (as determined by in situ hybridization (ISH), immunology and histology chemistry (IHC), western blot or by other means), trastuzumab resistance, tumour implantation method and implantation site, number or volume of implanted tumour cells, use of estradiol (dose, route of administration, delay to treatment). Missing data were requested from study authors. We extracted trastuzumab dose as mg/kg. Where this was quoted as μg, we estimated a weight-adjusted dose based on a typical average mouse weight of 25g.

### Risk of bias

Two review authors independently assessed the risk of bias in included studies by considering the following characteristics: peer-reviewed publication, and reporting of random allocation of tumour-bearing animal to treatment or control groups, blinded assessment of outcome, sample size calculation, statement of potential conflict of interests, compliance with animal welfare policy, explanation of rationale for disease model used, or multiple tumour models used, standardised number or volume of tumour cells implanted, reported number of animals in which the tumour did not grow, reporting and explanation of excluded animals, presentation of evidence that trastuzumab acts directly against the tumour, and consistent implantation site. We collated these into a quality checklist, similar to that described previously [21].

### Analysis

#### 1) Volume data

We initially intended to log transform the dataset (to account for exponential tumour growth) and then generate a normalized mean difference summary statistic (see protocol). However, through late 2014-early 2015 (after the data extraction phase) we observed that this produced nonparametrically distributed data and, with a large number of studies reporting improvements greater than +90%, there was a resultant skew with a large number of studies clustered close to the ceiling efficacy of +100%. This effect persisted even if the data were log transformed as planned *a priori*. Following discussion we instead decided to use a volume ratio summary statistic, as when log-transformed this would produce parametrically distributed data without ceiling. We elected to adopt the method suggested by Higgins et al [25]. We have expressed volume ratio values as the inverse log of these summary estimates to make the data easier to understand, and present these such that values over 1 favour the treatment group to make interpretation of figures more intuitive.

We used DerSimonian and Laird random effects meta-analysis to pool studies and stratified meta-analysis to search for sources of heterogeneity. For each group we again present the inverse log to give more intuitive values for mean and SE. As we have stratified the volume data 13 times, we used Bonferroni correction to give a critical value for significance of p<0.00385.

#### 2) Survival data

We generated an effect score of median survival ratio by dividing the treated group by control group. We then log-transformed the data and used a modified form of DerSimonian and Laird meta-analysis (weighted by the number of animals in the study instead of inverse variance) to generate a global efficacy estimate [26]. We used stratified meta-analysis as above.

#### 3) Publication bias

We used funnel plots, Egger regression and Trim and Fill analysis [27] to search for evidence of publication bias.

## Results

### Identification of papers

**Fig 1:**
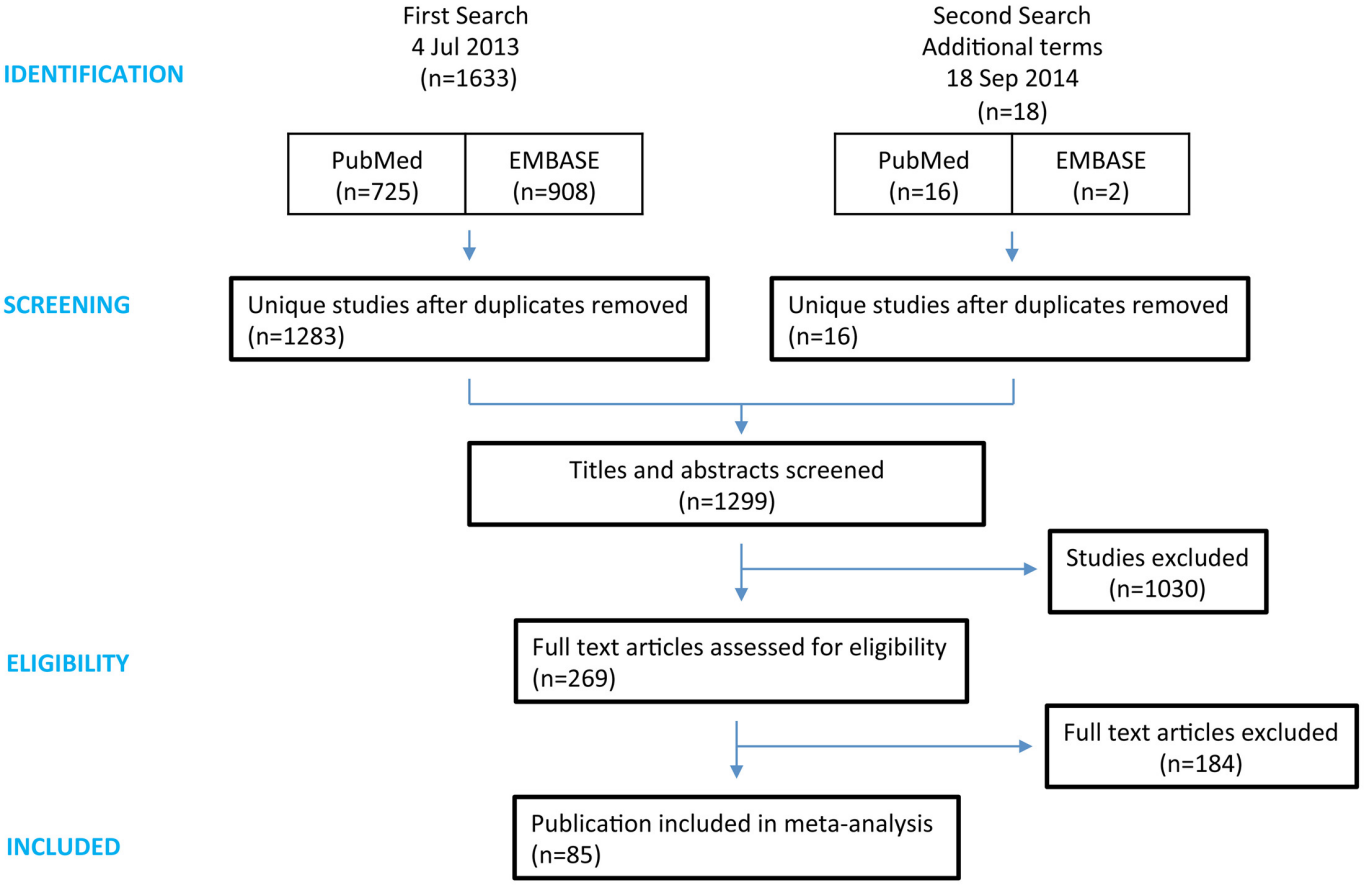
PRISMA flowchart of study selection. The first search was performed on 4 July 2013, using the terms relating to (breast tumor OR breast cancer) AND (trastuzumab OR Herceptin) (See S1 Table). On 18 September 2014, we added three further terms to expand the search with previous names of trastuzumab: (breast tumor OR breast cancer) AND ("rhuMAb HER2" OR "Anti-p185HER2 Monoclonal Antibody" OR "muMAb 4D5”).

We identified 1651 publications (PubMed 741, EMBASE 910), with 1299 articles remaining after duplicate removal. After title and abstract screening, full texts of 269 articles were retrieved. We found 85 studies reporting outcome as either median survival or tumour volume, but one study was excluded because tumour size was only inferred by measuring the activity of secreted molecules in the blood; tumour volume was measured directly in other studies. A further experiment was excluded during the analysis stage as the control group was treated with androstenedione, an androgen known to affect breast tumour growth in its own right [28]. Thus we included 83 studies reporting 169 experiments using 2076 mice in our meta-analysis (Fig 1). No experiments used other species (See S2 Table).

We have generated a spreadsheet containing the raw data extracted from each publication and published this on Figshare for public access (https://figshare.com/articles/Trastuzumab_preclinical_SRMA_-_raw_dataset/3406513).

Of these, 158 experiments assessed tumour volume as the outcome measure, with 11 reporting animal survival. Trastuzumab treatment was associated with a substantial reduction in tumour growth—with control tumours growing to 3.07 times the volume of treated tumours (95%CI 2.62–3.60). That is, tumour volume in treated animals was reduced by 67.4% (95%CI 61.8% - 72.2%). We observed considerable between-study heterogeneity (χ^2^ = 11376, df = 157, p<0.00385; I^2^ = 98.6%). Median survival was prolonged by a factor of 1.45 (1.30–1.62) although we did not observe any between-study heterogeneity in this smaller dataset (χ^2^ = 6.0, df = 10, p>0.05). Consequently we did not conduct a stratified meta-analysis of survival data.

### Risk of bias

**Fig 2:**
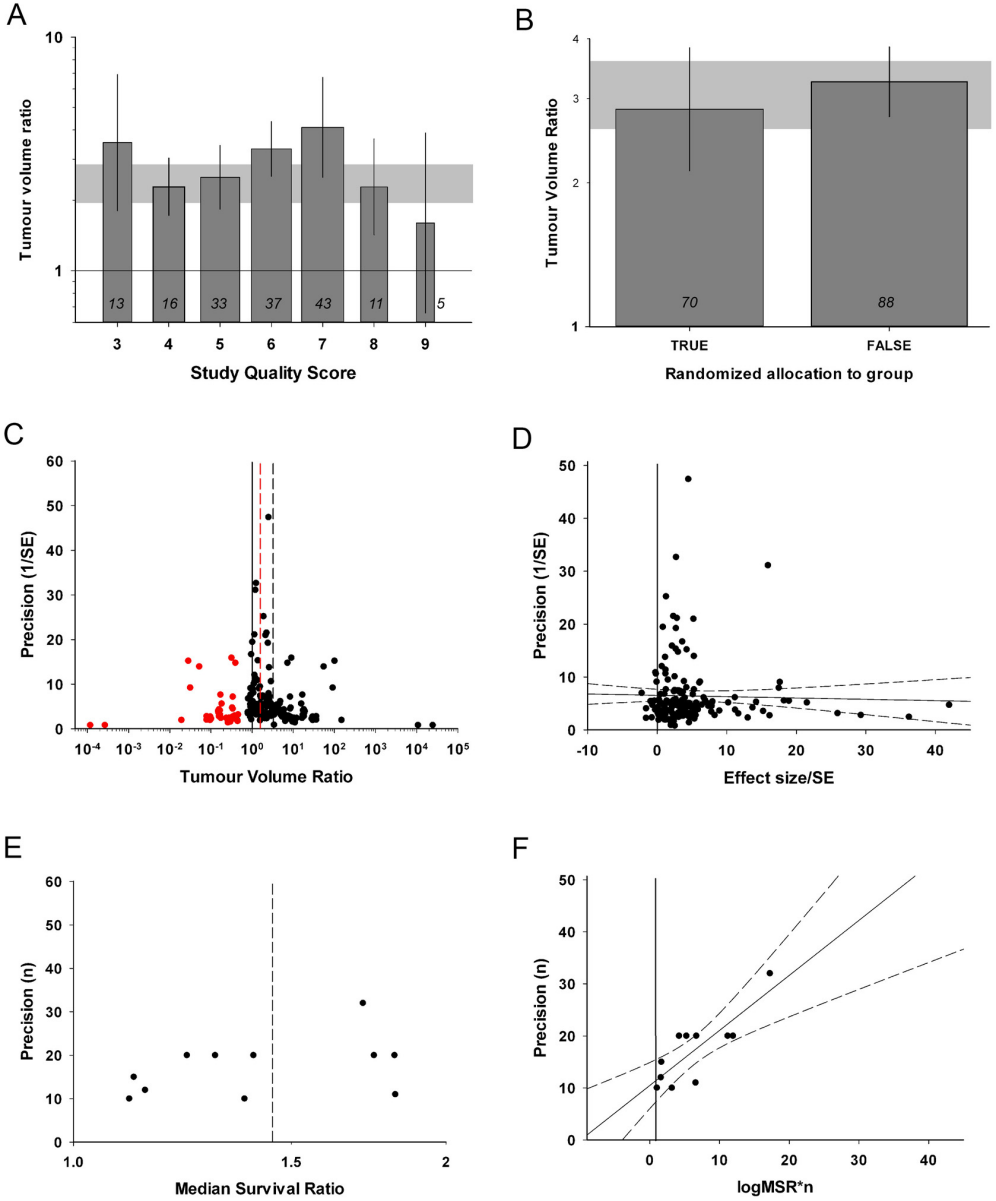
Evaluation of study quality and publication bias. (A) Quality score is associated with between-study heterogeneity tumour volume studies, with a trend for higher quality studies to report smaller effect sizes (χ^2^ = 1373, df = 6, p<0.00385). (B) Randomized studies were associated with smaller tumour volume reductions than non-randomised studies (χ^2^ = 68.1, df = 1, p<0.00385). (C and D) Both funnel plot and Egger regression indicate a presence of publication bias in tumour volume data (t = 8.772, p<0.001). Trim and Fill analysis added 38 ‘missing’ studies, with a sizeable reduction in global efficacy estimate (red plots). (E and F) For survival studies, funnel plot does not show any obvious asymmetry, but Egger regression suggests a publication bias (t = 8.772, p<0.001). The grey bands in A and B represent global 95% confidence intervals; columns represent mean ± 95%CI and column width a measure of number of comparisons within each stratum; the number of comparisons is included at the base of each column. Dotted lines in C and E represent global estimates of efficacy before (grey) and after (red) Trim and fill analysis. Dotted lines in D and F represent 95%CI of the regression. The vertical solid lines in A, C, D and F represent the level of neutral treatment effect.

Study quality was evaluated based on a modified 12-point checklist [21] with a median quality score of 6 (IQR 5–7; range 3 to 9. S3 Table). All of the 83 papers were published in a peer-reviewed journal, 36 (43%) reported random allocation to group, 36 (43%) stated whether a potential conflict of interest existed, and 31 (37%) described compliance with animal welfare policy. 49 (59%) studies compared more than one model, and 69 (83%) presented evidence that trastuzumab acts directly against the tumour. Standardized number or volume of tumour cells implanted was reported in 78 (94%) studies, and implantation site was consistent in 71 (86%) of studies. The number of animals in which the tumour did not grow was reported in only 4 (5%) studies and explanation of excluded animals was recorded in 12 (14%) studies. Surprisingly, none of the studies described the blinded outcome assessment or reported a sample size calculation. Stratification by the number of study quality checklist items scored accounted for significant heterogeneity between studies (χ^2^ = 1373, df = 6, p<0.00385), with higher quality studies reporting lower efficacy (Fig 2a). Similarly, studies that randomized group allocation reported lower efficacy (χ^2^ = 68.1, df = 1, P<0.00385. Fig 2b).

We also found substantial evidence of publication bias indicative of an overestimation of observed efficacy and implying an absence of small, inefficacious studies in the literature. Inspection of a funnel plot for tumour volume data suggests an excess of imprecise studies reporting high efficacy; there were only 14 experiments with results favouring control and of these, 9 reported volume ratios between 0.9 and 1 (Fig 2c). Egger regression was again consitent with publication bias (t = 8.772, p<0.001; Fig 2d) and Trim and Fill analysis suggests the presence of 38 ‘missing studies’ (red plots, Fig 2c). After inclusion of these imputed missing studies the adjusted global efficacy estimate was reduced from 3.07 to 1.77 (95%CI 1.46–2.14). For survival studies, the funnel plot did not show any obvious asymmetry (Fig 2e), although the presence of publication bias was suggested by a positive intercept on Egger regression (t = 5.340, p<0.001; Fig 2f). Trim and fill did not impute any missing studies for survival data. Importantly, we would expect some of the studies reported here to be neutral. For instance, some studies tested effciacy in cells known to have limited or no sensitivity to trastuzumab. The presence of such studies is therefore likely to lead to an underestimation of the scale of publication bias.

**Fig 3:**
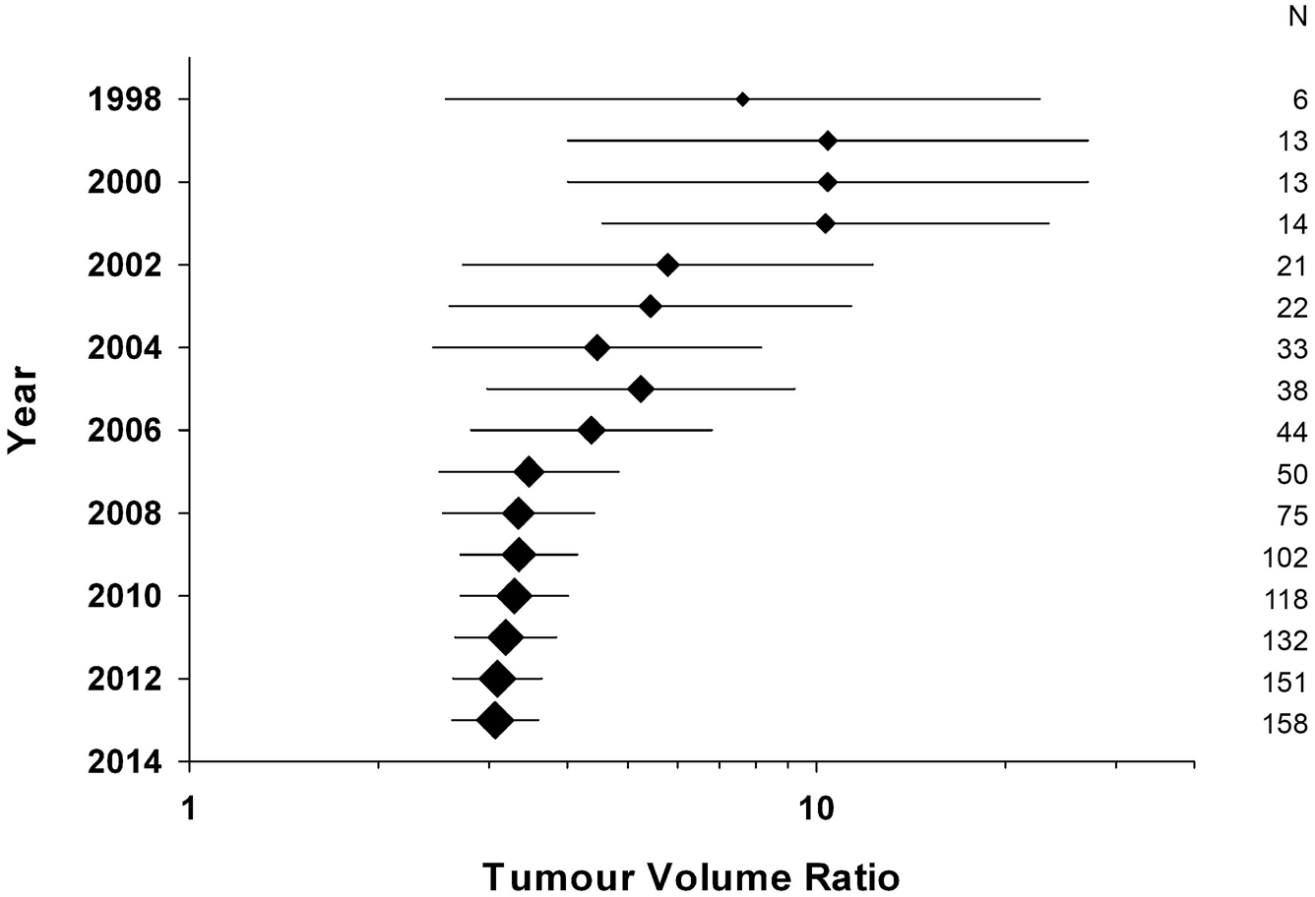
Cumulative meta-analysis. Plotting cumulative global efficacy estimate with time shows a steady trend for values to become more conservative with time. Plot size is representative of the number of studies, values for which are included on the right hand side, and error bars represent the 95%CI of the mean.

We have performed a cumulative meta-analysis for tumour volume to assess whether earlier studies might have been more influential in decisions relating to clinical trial design. Plotting global efficacy estimate for all studies published up to and including any given year shows a steady trend for values to become more conservative with time, with for instance a global efficacy estimate of 10.4 (4.01–27.1) in 2000 (Fig 3). This suggests a possible overestimation of efficacy during the earlier years of trastuzumab research, which may be due to differences in study design or quality measures. An alternative explanation would be if early studies tested efficacy in situations where we would expect it to be higher, for instance using tumour cell lines more sensitive to trastuzumab, or with intratumoural injection of drug. However, when we limit the analysis to tumour cell lines graded as ++ or +++ we see the same non-significant trend of decline in efficacy (S1 Fig)

### Animal and tumour models

**Fig 4:**
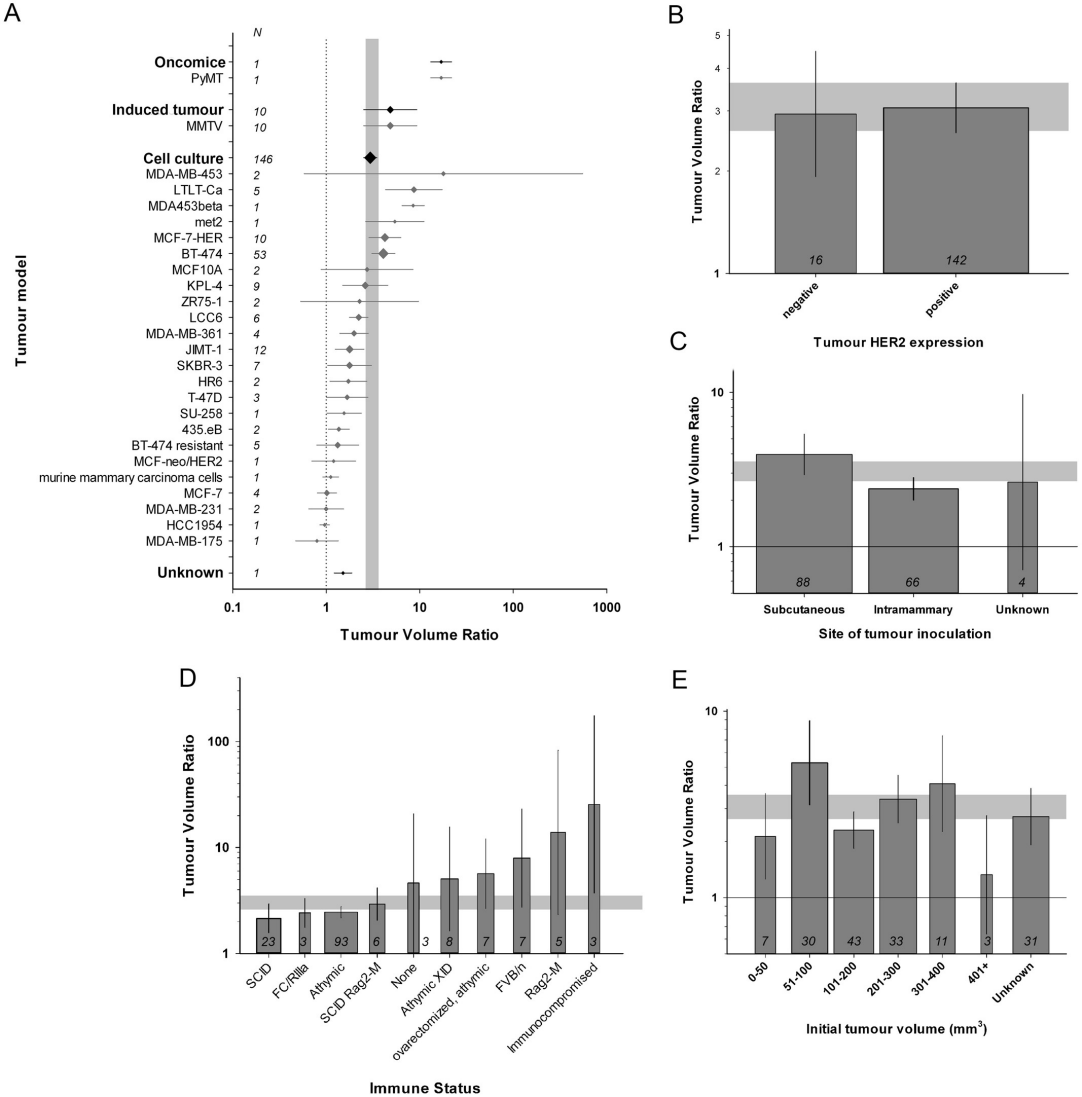
Stratification by animal and tumour model characteristics. (A) The specific xenograft model used were associated with a significant proportion of the between-study heterogeneity (χ^2^ = 4416, df = 23, P<0.00385). (B) Overexpression of HER2 in breast cancer tumours was associated with greater efficacy (χ^2^ = 160, df = 1, P<0.00385). (C) A greater reduction in tumour volume was observed with subcutaneous inoculation compared to intramammary route (χ^2^ = 810, df = 2, P<0.00385). (D) There is comorbidity related between-study heterogeneity (χ^2^ = 5130, df = 9, P<0.00385). The more commonly used athymic and SCID mice were associated with lower efficacies. (E) A significant proportion of between-study heterogeneity is accounted for by the tumour volume at treatment initiation, with a smaller treatment effect seen at either extreme (χ^2^ = 1042, df = 6, P<0.00385). The grey bands represent global 95% confidence intervals; diamonds (A) and columns (B-E) represent mean±95% CI and diamond size (A) column width (B-E) a measure of number of comparisons within each stratum; the number of comparisons is included inside the y-axis (A) and at the base of each column (B-E). The solid lines in A, C and E represent the level of neutral treatment effect.

The experimental breast cancer tumour models fell into three broad categories: tumour xenograft inoculation (146/158 volume studies, 11/11 survival studies), tumours induced using mouse mammary tumour virus (MMTV, 10/158), oncomice with a genetic predisposition to develop spontaneous tumours (1/158); and in 1 experiment the type of tumour was unclear. Efficacy was higher in experiments using oncomice and tumours induced by MMTV than those using xenografts (χ^2^ = 291, df = 3, p<0.00385). We observed 24 different tumour xenograft models, of which the BT-474 cell line was the most commonly used in both tumor volume (53/146) and survival studies (5/11). The specific xenograft model used were associated with a significant proportion of the observed heterogeneity in this group (χ^2^ = 4416, df = 23, p<0.00385. Fig 4a) and the MDA-MB-453 model appeared to be most sensitive to trastuzumab.

Tumours with augmented HER2 expression were commonly used (142/158 volume experiments, 11/11 survival). Overexpression of HER2 was linked to a better outcome (χ^2^ = 160, df = 1, p<0.00385, Fig 4b) when compared to HER2 negative or low expression. Furthermore, we found that studies reporting the use of tumour cells known to be trastuzumab-resistant *in vitro* were associated with smaller effect sizes (χ^2^ = 1009, df = 1, p<0.00385). Tumours were inoculated either subcutaneously (88/158 volume studies, 8/11 survival) or into mammary tissue (66/158; 3/11), and there was a greater reduction in tumour volume with subcutaneous inoculation than for tumours induced by intramammary inoculation (χ^2^ = 810, df = 2, p<0.00385; Fig 4c).

All studies included in our analysis used mice to evaluate trastuzumab efficacy. Almost all used mice with altered immune status (155/158 volume studies, 11/11 survival studies), most frequently athymic mice (for both volume (93/158) and survival studies (7/11)), with the next most common using severe combined immunodeficient (SCID) models (23/158 volume, 3/11 survival). These models differed in the observed efficacy (χ^2^ = 5130, df = 9, P<0.00385, Fig 4d), with athymic and SCID mice being associated with lower efficacies than animals with non-specific ‘immunocompromised’ mice or Rag2-M phenotype and those with no reported comorbidity. We included ovarectomy within this comorbidity stratification and efficacy was higher in athymic mice that were ovarectomized than in athymic mice that were not (see Fig 4d).

We used tumour volume at initiation of therapy as a surrogate for the delay to trastuzumab treatment, stratifying studies into groups of 0–50, 51–100, 101–200, 201–300, 301–400 and >400 mm^3^, as well as unknown. For studies providing this information, the majority started treatment when tumour volume was between 51 and 300 mm^3^. A significant proportion of between-study heterogeneity in volume data is accounted for by this stratification, with a smaller treatment effect seen at either extreme (χ^2^ = 1042, df = 6, p<0.00385, Fig 4e). This information was only available for 6 survival studies.

### Treatment regimen

To study the influence of different trastuzumab dosing regimens we stratified studies by total trastuzumab dose, by route of delivery and by tumour volume at treatment initiation as discussed above. We did not include treatment duration, administration frequency, number of cycles or other treatment details because specifying optimal dosing regimens in animals was not an aim of this study.

**Fig 5:**
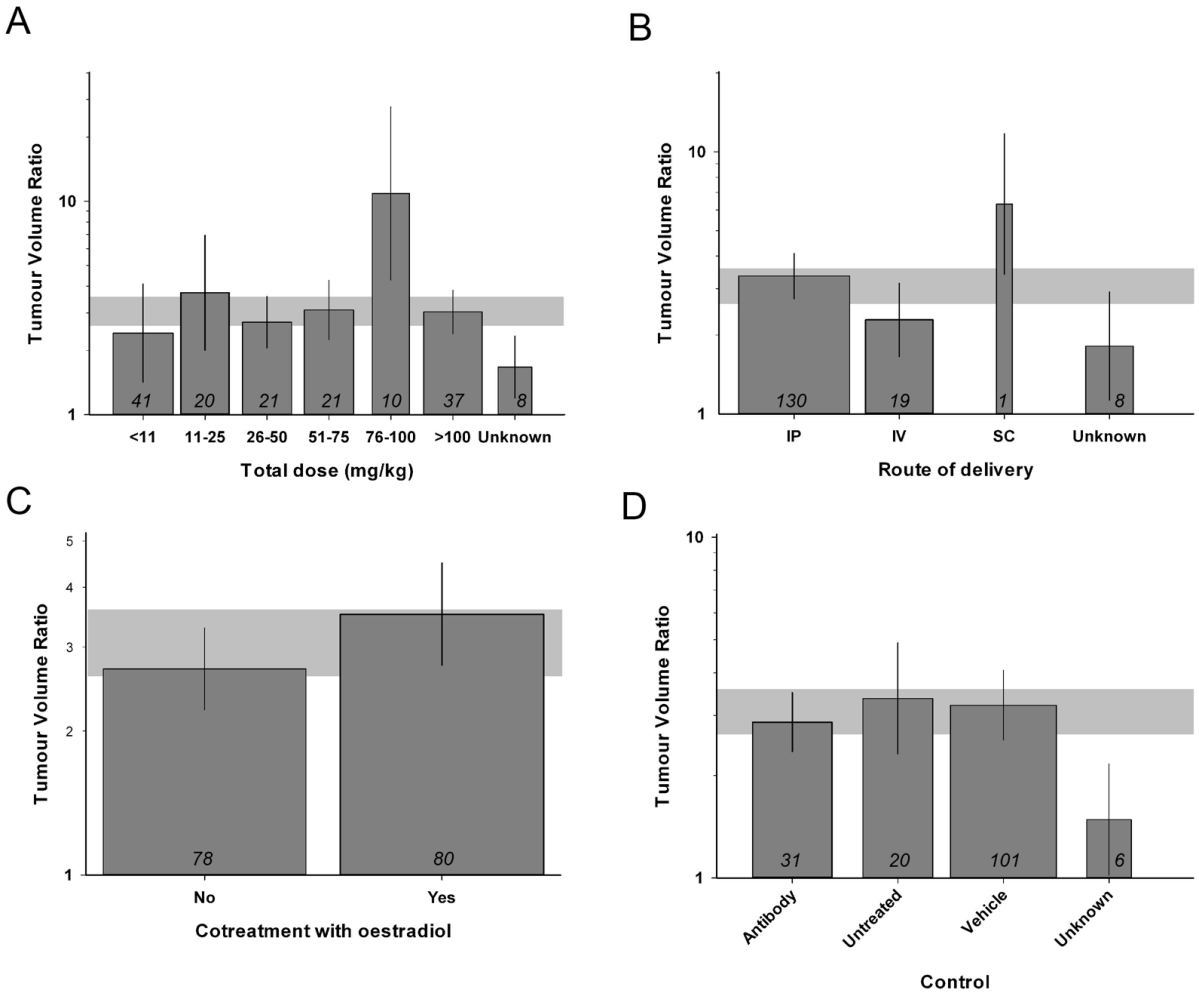
Stratification by trastuzumab treatment characteristics. Total trastuzumab dose (A. χ^2^ = 723, df = 6, P<0.00385), route of drug delivery (B. χ^2^ = 62, df = 3, P<0.00385), cotreatment with oestradiol (C. χ^2^ = 336, df = 1, P<0.00385) and the choice of control (D. χ^2^ = 956, df = 3, P<0.00385), accounted for significant between-study variability in reduction of tumour volume. The grey band represents global 95% confidence intervals; columns represent mean ± 95% CI and column width a measure of number of comparisons within each stratum; the number of comparisons is included at the base of each column.

We stratified the total trastuzumab dose into groups of <11, 11–25, 26–50, 51–75, 76–100 and >100 mg/kg, as well as unknown. Higher doses of trastuzumab were associated with greater efficacy (χ^2^ = 723, df = 6, P<0.00385, Fig 5a). We observed heterogeneity between different routes of trastuzumab delivery; intraperitoneal injection was most frequently used (130/158 volume and 9/11 survival studies). While all were effective, the single study reporting subcutaneous injection reported greater reductions in tumour volume than pooled estimates for other routes (χ^2^ = 62, df = 3, p<0.00385, Fig 5b).

Around half of the studies concomitantly implanted intradermal sustained-release estradiol pellets before xenograft inoculation (80/158 tumour volume studies, 4/11 survival studies), and these found greater reduction of tumour volume (χ^2^ = 336, df = 1, p<0.00385, Fig 5c). We also found that the choice of control was associated with between-study heterogeneity, in that studies reporting the use of non-functioning antibodies as control were associated with more conservative estimates of efficacy than those using vehicle or non-treated controls (χ^2^ = 956, df = 3, P<0.00385, Fig 5d).

## Discussion

In this review of 83 publications involving 169 experiments and 2076 animals, trastuzumab monotherapy appears to reduce tumour volume and prolong survival in animals bearing breast tumours. We have identified a large degree of between-study heterogeneity within the volume dataset but not across the smaller number of survival studies; and we have identified a number of features pertaining to both study quality and design that are associated with between-study heterogeneity. We have identified the presence of publication bias in both datasets.

In the tumour volume dataset we have demonstrated substantial between-study heterogeneity; furthermore, all the variables we tested accounted for between-heterogeneity in stratified meta-analysis. Our strategy is limited in its univariate nature, and does not account for covariance between the variables. [22, 29]. Colinearity might be taken into account using multivariate techniques such as a multi-variable meta-regression but this approach is seldom used in meta-analyses of animal data and its validity in this context is not known. The stratification accounting for the largest degree of heterogeneity (and therefore the first candidate variable for a multivariable approach) was the tumour model used; the large number of models (27, of which 16 were only used in 3 experiments or fewer) precludes multivariate analysis. We did not see between-study heterogeneity in the analysis of survival experiments. This may be due to the small number of studies, each of small size with low power, or may relate to limitations in the analysis brought about by the absence of data denoting within-study variance [26]. Consequently the results from our analysis should be considered hypothesis generating only and must be interpreted with caution.

We believe the preclinical trastuzumab literature is at high risk of bias. Study quality is modest, with a median score of 6 out of a possible 12 and no study met more than 9 checklist items. Less than half the studies reported randomized group allocation and no studies reported the blinded assessment of outcome or the use of a sample size calculation. We have observed lower efficacy in those studies at lower risk of bias—namely studies achieving a higher quality score and those randomizing—and this phenomenon is well documented in the preclinical neurosciences literature [22, 23]. Furthermore there is considerable publication bias amongst both datasets with a grossly asymmetrical funnel plot for tumour volume data and positive Egger’s test for both datasets. Even Trim and Fill, a conservative technique [30], led to an adjusted estimate of efficacy almost 50% lower than the unadjusted figure, implying a large excess of small studies reporting large efficacy in the published literature.

Optimising study quality is important as overstated results from low quality animal studies can misinform decisions to proceed to clinical trial [31] and inaccurate conclusions from these data will inevitably lead to unnecessary animal suffering. There is increasing awareness of this phenomenon and quality is in general improving with time [32]. Nonetheless we believe that a standardized report pattern including measures to reduce risk of bias must be widely adopted for animal research to continue to be valid as a translational strategy [33]. An exemplar reporting framework is the ARRIVE guidelines [33, 34].

The first article evaluating the efficacy of trastuzumab on breast cancer animal model was published in 1991 [35]. However, this article was not included in our analysis as the primary outcome was tumour weight and was therefore did not meet our inclusion criteria. The first positive clinical study of trastuzumab showed objective responses in 43 of 46 patients with tumours overexpressing HER2 in 1996 [36]; but the earliest animal study included in our analysis was published in 1998. There is a trend for reported effect size to become more conservative with time, implying that the problem of overstated efficacy was larger at the very time when this information was most likely to be applied to human trials. Efficacy estimates appear to decrease in magnitude from 2001: this may coincide with attempts to characterise trastuzumab resistance, a process utilising different animal models, dosing regimens and trastuzumab-resistant xenografts [37].

There are several factors that may influence an experimenter’s choice of tumour model [38, 39]. We have found that this choice was the largest source of heterogeneity and, when taken with similar findings in glioma studies [21, 23], makes model choice a major determinant of study outcome in cancer studies. This was why we included a justification for tumour model choice or the testing of two or more tumour models as a study quality criterion. BT-474 and MCF-7 (HER2-transfected) were the most popular cell lines and these appeared to be amongst the more trastuzumab-sensitive.

We have also shown that the use of immunocompromised mice is the default *modus operandi* for preclinical breast cancer studies. While this is clearly important for the successful inoculation of xenografts it should be borne in mind that the immune system plays a key role in the body’s response to cancer and contributes substantially to the therapeutic effects of monoclonal antibodies, [40, 41] this will not be recapitulated in studies using immunodeficient animals. Greater volume reduction was seen in tumours inoculated subcutaneously than those into mammary fat pad. Since the host environment in the latter more accurately represents that in human disease we recommend that future studies should adopt this approach where possible.

As for control reagents, we noticed a slightly reduced efficacy in studies controlling with antibody (mainly with non-specific immunoglobulin), compared to vehicle and untreated controls. While it may be possible for non-functioning antibodies to modulate the body’s response to tumour this observation may also reflect the association between study quality and outcome alluded to above.

In human disease, amplification of HER2 is usually associated with worse prognosis in breast cancer; and trastuzumab is generally more effective against tumours overexpressing HER2 to a higher degree. It is therefore relevant to assess HER2 status in order to prognosticate treatment [42, 43]. This is generally done by measuring HER2 gene copy number with in situ hybridization (ISH) or immunohistochemistry (IHC) [5, 9]. In our analysis we dichotomised HER2 expression status into negative, with expression reported as low or negative, and positive, with HER2 overexpressed, observing greater efficacy in tumours overexpressing HER2. We had initially intended to compare efficacy between different degrees of HER2 overexpression, however we found that studies used a range of different methods to quantify this, including but not limited to IHC and ISH, and in 65 experiments the degree of HER2 overexpression was not stated. Therefore this stratification was not possible.

The timing and dosage of treatment for any condition are inherently important and a wide range of nuanced regimens were reported in the literature. However, characterizing details of this were not the primary aims of this study. We have included analyses of total dose and delay to treatment to gain a superficial insight into the regimens used and to give some simple comparisons between types. We have observed what seems to be a dose-response relationship with trastuzumab although side effect profile has not been accounted for due to the lack of information reported in the studies. Tumour volume reduction was relatively consistent with moderate tumour initial volumes, with lower efficacy with larger tumours. To go further than this would require prospective experiments directly comparing specific treatment regimens at various stages of disease. Animal studies in general used intraperitoneal drug delivery, differing from the intravenous route favoured in clinical practice. We saw greater efficacy in the single study reporting subcutaneous delivery; subcutaneous administration has been tested in humans with the advantage of convenience [44, 45], although there may be more serious adverse effects than with intravenous delivery [46].

Hormone level plays an essential role in initiation, promotion, and progression of breast cancer [47, 48]. Estrogen is required for the normal development of the mammary gland and is predominantly produced in the ovary. Several studies reported the use of ovarectomy to simulate the postmenopausal, oestrogen-deplete, state. In these studies all animals were also athymic and trastuzumab efficacy was greater in this group than in the athymic (non-ovarectomized) animals (see Fig 4d and S2 Table). There is some evidence from animal models showing that estrogens are breast carcinogens [49, 50] and when inoculating tumour xeongrafts, concordant oestrogen delivery could increase the chance of tumour growth [51]. Estrogen was used as a cotreatment in about half of the comparisons in our analyses while establishing breast cancer model, mostly via a subcutaneous injection of sustained release estradiol less than a week prior to tumour cell inoculation. Cotreatment resulted a higher efficacy in tumour volume studies. This may reflect variations in tumour model or the fact that tumours co-expressing HER2 and estrogen receptors are associated with a better outcome than those expressing HER2 alone [52]

To summarise, we have found trastuzumab to be effective in animal breast cancer models across a range of experimental circumstances, correlating with clinical experience. There is evidence to suggest this literature is at high risk of bias, through the presence of publication bias and low prevalence of randomization and blinding. We have also defined a number of study design features that appear to affect the perceived efficacy of trastuzumab. This meta-analysis is of course limited, especially in the context of low-quality underlying data and the likely presence of colinearity, so these results should be interpreted with caution. We hope that our conclusions may help to streamline future research in experimental breast cancer and in the wider preclinical setting.

## Supporting Information

S1 Fig Cumulative meta-analysis limited to tumour lines with high HER2 expression. (TIF)

S1 Table Search strategy summary. (DOCX)

S2 Table Study characteristics. (DOCX)

S3 Table Study quality scores. (DOCX)

S4 Table PRISMA checklist. (DOC)

S5 Table Raw data. (XLS)

S1 Text Study protocol. (PDF)
