# Supplementary material for: The Efficacy of Trastuzumab in Animal Models of Breast Cancer: A Systematic Review and Meta-Analysis
Source: PLoS One. 2016 Jul 27;11(7):e0158240. doi: 10.1371/journal.pone.0158240 (PMC4963137; doi:10.1371/journal.pone.0158240)
Supplement: S2 Table — (DOCX) [file pone.0158240.s003.docx]

Supplementary Material S2: Study Characteristics

| Name | Year | Group | Quality Score | Animal | Species | Breast Cancer Model | HER2 Level | Resistant Tumour *in vitro?* | Cotreatment with Estradiol | Number in Control Group | Number in Treatment Group | Control Agent | Route of Trastuzumab Delivery | Outcome Measure | Tumour Volume Ratio (Rx/C) | lower 95%CI | upper 95%CI |
| --- | --- | --- | --- | --- | --- | --- | --- | --- | --- | --- | --- | --- | --- | --- | --- | --- | --- |
| Abbas, N.([Abbas et al., 2011](#_ENREF_1)) | 2011 |  | 6 | Mouse | Balb/C nu/nu (NCR) | SKBR-3 | 3+ | No | No | 10 | 5 | Vehicle | IVenous | Survival | 1.11 | - | - |
| Agus, D. B.([Agus et al., 2002](#_ENREF_2)) | 2002 | A | 4 | Mouse | nude athymic BALB/c | BT-474 | overexpressed, level unknown | No | Yes | 6 | 6 | Vehicle | IPeritoneal | Volume | 0.92 | 0.83 | 1.03 |
|  |  | B | 4 | Mouse | nude athymic BALB/c | MCF-7 | 1+ | No | Yes | 6 | 6 | Vehicle | IPeritoneal | Volume | 4.92 | 4.18 | 5.79 |
| Anido, J.([Anido et al., 2006](#_ENREF_3)) | 2006 | A | 5 | Mouse | BALB/c athymic (nu+/nu+) | T-47D HER2 | 3+ | No | Yes | 6 | 6 | Vehicle | IPeritoneal | Volume | 1.06 | 0.85 | 1.32 |
|  |  | B | 5 | Mouse | BALB/c athymic (nu+/nu+) | T-47D HER2 ∆Nar I | 1+ | No | Yes | 6 | 6 | Vehicle | IPeritoneal | Volume | 2.37 | 1.78 | 3.15 |
| Arnal-Estape, A.([Arnal-Estape et al., 2010](#_ENREF_4)) | 2010 | A | 6 | Mouse | Nude-beige (NIH-bg-nu-xidBR;  NCI) | (RAS/ERBB2/F-LIP)-expressing MCF10A | overexpressed, level unknown | Yes | No | 7 | 7 | Vehicle | IPeritoneal | Volume | 1.59 | 1.39 | 1.82 |
|  |  | B | 6 | Mouse | Nude-beige (NIH-bg-nu-xidBR;  NCI) | (RAS/ERBB2)-expressing MCF10A | 2+ | No | No | 7 | 7 | Vehicle | IPeritoneal | Volume | 5.06 | 3.66 | 6.99 |
| Barok, M.([Barok et al., 2008](#_ENREF_5)) | 2008 |  | 3 | Mouse | SCID C.B-17  scid/scid | JIMT-1 | overexpressed, level unknown | Yes | No | 8 | 8 | Vehicle | IPeritoneal | Volume | 2.51 | 2.10 | 3.00 |
| Barok, M.([Barok et al., 2007](#_ENREF_6)) | 2007 | A | 4 | Mouse | SCID C.B-17scid/scid | JIMT-1 | overexpressed, level unknown | Yes | No | 8 | 4 | Vehicle | IPeritoneal | Volume | 1.39 | 1.04 | 1.85 |
|  |  | B | 4 | Mouse | SCID C.B-17scid/scid | JIMT-1 | overexpressed, level unknown | Yes | No | 8 | 8 | Vehicle | IPeritoneal | Volume | 1.48 | 1.12 | 1.96 |
|  |  | C | 4 | Mouse | SCID C.B-17scid/scid | JIMT-1 | overexpressed, level unknown | Yes | No | 8 | 4 | Vehicle | IPeritoneal | Volume | 1.66 | 1.37 | 2.03 |
|  |  | D | 4 | Mouse | SCID C.B-17scid/scid | JIMT-1 | overexpressed, level unknown | Yes | No | 8 | 8 | Vehicle | IPeritoneal | Volume | 8.80 | 7.13 | 10.88 |
| Baselga, J.([Baselga et al., 1998](#_ENREF_7)) | 1998 | A | 3 | Mouse | BALB/c nude | BT-474 | overexpressed, level unknown | No | Yes | 6 | 6 | Antibody | IPeritoneal | Volume | 1.57 | 1.29 | 1.91 |
|  |  | B | 3 | Mouse | BALB/c nude | BT-474 | overexpressed, level unknown | No | Yes | 6 | 6 | Antibody | IPeritoneal | Volume | 2.52 | 1.85 | 3.45 |
|  |  | C | 3 | Mouse | BALB/c nude | BT-474 | overexpressed, level unknown | No | Yes | 6 | 6 | Antibody | IPeritoneal | Volume | 7.82 | 5.04 | 12.11 |
|  |  | D | 3 | Mouse | BALB/c nude | BT-474 | overexpressed, level unknown | No | Yes | 11 | 10 | Antibody | IPeritoneal | Volume | 10.46 | 6.92 | 15.81 |
|  |  | E | 3 | Mouse | BALB/c nude | BT-474 | overexpressed, level unknown | No | Yes | 11 | 10 | Antibody | IPeritoneal | Volume | 18.38 | 12.82 | 26.36 |
|  |  | F | 3 | Mouse | BALB/c nude | BT-474 | overexpressed, level unknown | No | Yes | 11 | 8 | Antibody | IPeritoneal | Volume | 36.74 | 25.62 | 52.68 |
| Beyer, I.([Beyer et al., 2011](#_ENREF_8)) | 2011 |  | 5 | Mouse | CB17 SCID-beige | HCC1954 | +- | No | No | 5 | 5 | Vehicle | IPeritoneal | Volume | 0.96 | 0.90 | 1.02 |
| Bocangel, D.([Bocangel et al., 2006](#_ENREF_9)) | 2006 |  | 4 | Mouse | athymic nude | MCF-7-Her-18 | overexpressed, level unknown | No | Yes | 6 | 6 | Vehicle | Unknown | Volume | 2.53 | 2.47 | 2.58 |
| Brodie, A.([Brodie et al., 2007](#_ENREF_10)) | 2007 |  | 4 | Mouse | ovarectomized, BALB/c athymic nude | LTLT-Ca | 2+ | No | No | 3 | 3 | Untreated | SubCut | Volume | 6.32 | 4.61 | 8.67 |
| Capietto, A. H.([Capietto et al., 2011](#_ENREF_11)) | 2011 | A | 7 | Mouse | SCID/Beige | SKBR-3 | 3+ | No | No | 5 | 5 | Vehicle | IPeritoneal | Volume | 0.97 | 0.60 | 1.56 |
|  |  | B | 7 | Mouse | SCID/Beige | SKBR-3 | 3+ | No | No | 5 | 5 | Vehicle | IPeritoneal | Volume | 1.30 | 1.06 | 1.61 |
| Chakrabarty, A.([Chakrabarty et al., 2013](#_ENREF_12)) | 2013 |  | 6 | Mouse | athymic | HR6 | overexpressed, level unknown | Yes | Yes | 3 | 3 | Vehicle | IPeritoneal | Volume | 3.47 | 1.13 | 10.65 |
| Chakrabarty, A.([Chakrabarty et al., 2012](#_ENREF_13)) | 2012 |  | 7 | Mouse | athymic | BT-474 | overexpressed, level unknown | No | Yes | 8 | 8 | Vehicle | IPeritoneal | Volume | 7.91 | 5.58 | 11.23 |
| Chan, C. H.([Chan et al., 2012](#_ENREF_14)) | 2012 | A | 5 | Mouse | athymic nude | BT-474-M1 cells with luciferase knockdown (shLuc) | overexpressed, level unknown | No | No | 6 | 6 | Antibody | IPeritoneal | Volume | 2.14 | 2.04 | 2.24 |
|  |  | B | 5 | Mouse | athymic nude | BT-474-M1 cells with Skp2 knockdown (shSkp2) | overexpressed, level unknown | No | No | 6 | 6 | Antibody | IPeritoneal | Volume | 18.36 | 14.58 | 23.13 |
| Lai, H. W.([Lai et al., 2012](#_ENREF_43)) | 2012 |  | 5 | Mouse | athymic nude | BT-474 | 3+ | No | Yes | 6 | 6 | Vehicle | IPeritoneal | Volume | 8.52 | 6.81 | 10.65 |
| Cheung, N. K.([Cheung et al., 2002](#_ENREF_15)) | 2002 |  | 3 | Mouse | nude (NCI) | BT-474 | overexpressed, level unknown | No | Yes | 4 | 9 | Untreated | IVenous | Volume | 1.24 | 1.01 | 1.51 |
| Ching, C. L.([Ching et al., 2009](#_ENREF_16)) | 2009 | A | 8 | Mouse | athymic nu/nu | JIMT-1 | 3+ | Yes | Yes | 9 | 9 | Untreated | IPeritoneal | Volume | 0.92 | 0.75 | 1.13 |
|  |  | B | 8 | Mouse | athymic nu/nu | JIMT-1 | 3+ | Yes | Yes | 10 | 10 | Untreated | IPeritoneal | Volume | 0.94 | 0.83 | 1.08 |
|  |  | C | 8 | Mouse | athymic nu/nu | JIMT-1 | 3+ | Yes | Yes | 9 | 9 | Untreated | IPeritoneal | Volume | 0.99 | 0.81 | 1.20 |
|  |  | D | 8 | Mouse | athymic nu/nu | BT-474 | 3+ | No | Yes | 10 | 10 | Untreated | IPeritoneal | Volume | 1.24 | 1.03 | 1.50 |
|  |  | E | 8 | Mouse | athymic nu/nu | JIMT-1 | 3+ | Yes | Yes | 10 | 10 | Untreated | IPeritoneal | Volume | 3.06 | 2.50 | 3.74 |
| Chiu, G. N.([Chiu et al., 2007](#_ENREF_17)) | 2007 |  | 3 | Mouse | Rag2-M | LCC6-HER2 | overexpressed, level unknown | No | No | 6 | 6 | Vehicle | IVenous | Volume | 1.67 | 1.44 | 1.93 |
| Chumsri, S.([Chumsri et al., 2011](#_ENREF_18)) | 2011 |  | 3 | Mouse | ovarectomized, BALB/c athymic nude | MCF-7Ca | 1+ | No | No | 3 | 3 | Unknown | Unknown | Volume | 0.44 | 0.26 | 0.75 |
| Colbern, G. T.([Colbern et al., 1999](#_ENREF_19)) | 1999 | A | 7 | Mouse | Immunocompromised (Ncr.nu/nu） | MDA-MB-453 | 2+ | No | Yes | 12 | 12 | Vehicle | IPeritoneal | Volume | 2.90 | 2.64 | 3.19 |
|  |  | B | 7 | Mouse | Immunocompromised (Ncr.nu/nu） | BT-474 | 3+ | No | Yes | 12 | 12 | Vehicle | IPeritoneal | Volume | 55.57 | 51.72 | 59.70 |
|  |  | C | 7 | Mouse | Immunocompromised (Ncr.nu/nu） | BT-474 | 3+ | No | Yes | 12 | 12 | Vehicle | IPeritoneal | Volume | 102.28 | 95.76 | 109.24 |
| Costantini, D. L.([Costantini et al., 2010](#_ENREF_20)) | 2010 | A | 5 | Mouse | athymic CD-1 | MDA-MB-361 | 3+ | No | No | 6 | 6 | Vehicle | IPeritoneal | Volume & Survival | 0.91 | 0.63 | 1.32 |
|  |  | B | 5 | Mouse | athymic CD-1 | MDA-MB-231 | 1+ | No | No | 6 | 6 | Vehicle | IPeritoneal | Volume | 1.26 | 0.99 | 1.59 |
| Damiano, V.([Damiano et al., 2009](#_ENREF_21)) | 2009 | A | 7 | Mouse | BALB/cAnNCrlBR athymic (nu+/nu+) | JIMT-1 | 3+ | Yes | No | 10 | 10 | Unknown | IPeritoneal | Volume & Survival | 2.55 | 2.25 | 2.89 |
|  |  | B | 7 | Mouse | BALB/cAnNCrlBR athymic (nu+/nu+) | KPL-4 | 3+ | Yes | No | 10 | 10 | Unknown | IPeritoneal | Volume & Survival | 2.66 | 2.32 | 3.05 |
| Foy, K. C.([Foy et al., 2012](#_ENREF_22)) | 2012 |  | 3 | Mouse | FVB/n | PyMT | overexpressed, level unknown | No | No | 5 | 5 | Untreated | IVenous | Volume | 16.94 | 14.86 | 19.31 |
| Francia, G.([Francia et al., 2009](#_ENREF_23)) | 2009 |  | 6 | Mouse | SCID CB17 | met2 | overexpressed, level unknown | No | No | 5 | 6 | Vehicle | IPeritoneal | Volume & Survival | 5.41 | 3.75 | 7.81 |
| Fujimoto-Ouchi, K.([Fujimoto-Ouchi et al., 2002](#_ENREF_24)) | 2002 | A | 7 | Mouse | BALB/c-nu/nu | BT-474 | 3+ | No | Yes | 8 | 8 | Antibody | IPeritoneal | Volume | 1.52 | 1.17 | 1.97 |
|  |  | B | 7 | Mouse | BALB/c-nu/nu | KPL-4 | overexpressed, level unknown | No | No | 8 | 8 | Antibody | IPeritoneal | Volume | 2.04 | 1.70 | 2.44 |
| Fujimoto-Ouchi, K.([Fujimoto-Ouchi et al., 2010](#_ENREF_25)) | 2010 | A | 6 | Mouse | BALB/c-nu/nu | KPL-4 | overexpressed, level unknown | Yes | No | 8 | 16 | Antibody | IPeritoneal | Volume | 1.15 | 1.09 | 1.20 |
|  |  | B | 6 | Mouse | BALB/c-nu/nu | KPL-4 | overexpressed, level unknown | Yes | No | 8 | 16 | Antibody | IPeritoneal | Volume | 1.21 | 1.17 | 1.25 |
| Garrett, J. T.([Garrett et al., 2012](#_ENREF_26)) | 2012 | A | 8 | Mouse | athymic | BT-474 | 3+ | No | Yes | 8 | 8 | Vehicle | IPeritoneal | Volume | 1.67 | 1.32 | 2.12 |
|  |  | B | 8 | Mouse | athymic | HR6 | overexpressed, level unknown | Yes | Yes | 7 | 7 | Vehicle | IPeritoneal | Volume | 4.33 | 3.53 | 5.31 |
| Gee, M. S.([Gee et al., 2008](#_ENREF_27)) | 2008 | A | 7 | Mouse | C57BL/6 nude | BT-474 | 3+ | No | Yes | 5 | 10 | Vehicle | IPeritoneal | Volume | 1.21 | 1.00 | 1.47 |
|  |  | B | 7 | Mouse | C57BL/6 nude | BT-474 | 3+ | No | Yes | 5 | 5 | Vehicle | IPeritoneal | Volume | 1.37 | 1.14 | 1.65 |
|  |  | C | 7 | Mouse | C57BL/6 nude | SKBR-3 | 3+ | No | No | 5 | 5 | Vehicle | IPeritoneal | Volume | 3.77 | 2.91 | 4.89 |
|  |  | D | 7 | Mouse | C57BL/6 nude | MCF-7 | 1+ | No | Yes | 5 | 5 | Vehicle | IPeritoneal | Volume | 7.78 | 5.43 | 11.14 |
| Gee, M. S.([Gee et al., 2007](#_ENREF_28)) | 2007 | A | 6 | Mouse | C57BL/6J nude | SKBR-3 R1 | 3+ | Yes | No | 3 | 3 | Antibody | IPeritoneal | Volume | 1.19 | 0.98 | 1.44 |
|  |  | B | 6 | Mouse | C57BL/6J nude | SKBR-3 | 3+ | No | No | 3 | 3 | Antibody | IPeritoneal | Volume | 1.27 | 1.06 | 1.53 |
|  |  | C | 6 | Mouse | C57BL/6J nude | BT-474 | 3+ | No | Yes | 3 | 3 | Antibody | IPeritoneal | Volume | 3.58 | 2.89 | 4.43 |
|  |  | D | 6 | Mouse | C57BL/6J nude | MCF-7 | 1+ | No | Yes | 3 | 3 | Antibody | IPeritoneal | Volume | 7.18 | 4.77 | 10.81 |
| Gijsen, M.([Gijsen et al., 2010](#_ENREF_29)) | 2010 |  | 6 | Mouse | NMRI nude | BT-474 | overexpressed, level unknown | No | No | 8 | 8 | Vehicle | IPeritoneal | Volume | 4.65 | 3.54 | 6.11 |
| Han, H.([Han and Davis, 2013](#_ENREF_30)) | 2013 | A | 8 | Mouse | NCr nude | BT-474 | overexpressed, level unknown | No | Yes | 8 | 6 | Vehicle | IVenous | Volume | 3.48 | 2.53 | 4.79 |
|  |  | B | 8 | Mouse | NCr nude | BT-474 | overexpressed, level unknown | No | Yes | 8 | 8 | Vehicle | IVenous | Volume | 17.20 | 12.44 | 23.78 |
| Heyerdahl, H.([Heyerdahl et al., 2012](#_ENREF_31)) | 2012 | A | 9 | Mouse | Balb/c nu/nu (NCR) | SKBR-3 | overexpressed, level unknown | No | No | 4 | 3 | Vehicle | IVenous | Volume | 0.80 | 0.59 | 1.07 |
|  |  | B | 9 | Mouse | Balb/c nu/nu (NCR) | SKBR-3 | overexpressed, level unknown | No | No | 4 | 5 | Vehicle | IVenous | Volume | 1.05 | 0.89 | 1.23 |
| Inoue, S.([Inoue et al., 2011](#_ENREF_32)) | 2011 |  | 6 | Mouse | athymic | BT-474 | 3+ | No | Yes | 10 | 11 | Vehicle | IVenous | Volume | 3.48 | 2.54 | 4.77 |
| Ithimakin, S.([Ithimakin et al., 2013](#_ENREF_33)) | 2013 | A | 5 | Mouse | NOD/SCID | ZR75-1 | +- | No | No | 3 | 3 | Unknown | IPeritoneal | Volume | 1.05 | 0.94 | 1.18 |
|  |  | B | 5 | Mouse | NOD/SCID | BT-474 | 3+ | No | No | 3 | 3 | Unknown | IPeritoneal | Volume | 1.09 | 0.96 | 1.23 |
|  |  | C | 5 | Mouse | NOD/SCID | MCF-7 | +- | No | No | 3 | 3 | Unknown | IPeritoneal | Volume | 1.57 | 1.41 | 1.75 |
| Jerome, L.([Jerome et al., 2006](#_ENREF_34)) | 2006 |  | 5 | Mouse | CD1 (nu/nu) athymic | MCF-7-HER2-18 | 3+ | No | Yes | 7 | 7 | Vehicle | IPeritoneal | Volume | 1.28 | 1.05 | 1.56 |
| Jumbe, N. L.([Jumbe et al., 2010](#_ENREF_35)) | 2010 |  | 8 | Mouse | beige nude XID | BT474EEI | 3+ | Yes | No | 3 | 3 | Vehicle | IVenous | Volume | 1.43 | 1.19 | 1.72 |
| Junttila, T. T.([Junttila et al., 2009](#_ENREF_36)) | 2009 | A | 7 | Mouse | NCR nude | MDA-MB-361 | 1+ | Yes | Yes | 3 | 3 | Vehicle | IPeritoneal | Volume | 1.27 | 0.92 | 1.74 |
|  |  | B | 7 | Mouse | C.B-17/IcrHsd-Prkdc scid | BT474-M1 | overexpressed, level unknown | No | No | 3 | 3 | Vehicle | IVenous | Volume | 3.51 | 2.81 | 4.38 |
| Junttila, T. T.([Junttila et al., 2011](#_ENREF_37)) | 2011 | A | 6 | Mouse | NCR nude | MCF-neo/HER2 | 3+ | Yes | Yes | 10 | 10 | Vehicle | IVenous | Volume | 1.14 | 0.94 | 1.39 |
|  |  | B | 6 | Mouse | Fo5 | MMTV | overexpressed, level unknown | No | No | 10 | 10 | Vehicle | IVenous | Volume | 1.20 | 0.91 | 1.58 |
| Junttila, T. T.([Junttila et al., 2010](#_ENREF_38)) | 2010 | A | 7 | Mouse | FCγRIIIa | MCF-neo/HER2 | overexpressed, level unknown | No | Yes | 10 | 10 | Antibody | IVenous | Volume | 2.06 | 1.35 | 3.16 |
|  |  | B | 7 | Mouse | FcγRI−/−FcγRIII−/−RAG2−/−Tg (human FcγRIIIa) | KPL-4 | overexpressed, level unknown | No | No | 9 | 9 | Vehicle | IVenous | Volume | 2.28 | 1.48 | 3.52 |
|  |  | C | 7 | Mouse | FcγRI−/−FcγRIII−/−RAG2−/−Tg (human FcγRIIIa) | KPL-4 | overexpressed, level unknown | No | No | 9 | 9 | Vehicle | IVenous | Volume | 2.52 | 2.09 | 3.05 |
|  |  | D | 7 | Mouse | SCID-beige | KPL-4 | overexpressed, level unknown | No | No | 8 | 8 | Vehicle | IPeritoneal | Volume | 18.79 | 14.19 | 24.87 |
| Klos, K. S.([Klos et al., 2003](#_ENREF_39)) | 2003 |  | 4 | Mouse | ICR SCID | 435.eB | overexpressed, level unknown | No | No | 8 | 8 | Antibody | IPeritoneal | Volume | 1.46 | 1.20 | 1.77 |
| Kohrt, H. E.([Kohrt et al., 2012](#_ENREF_40)) | 2012 | A | 7 | Mouse | athymic nu/nu Foxn1nu | BT474M1 | 3+ | No | Yes | 10 | 10 | Antibody | IPeritoneal | Volume & Survival | 1.48 | 1.33 | 1.65 |
|  |  | B | 7 | Mouse | athymic nu/nu Foxn1nu | BT474M1 | 3+ | No | Yes | 10 | 10 | Antibody | IPeritoneal | Volume & Survival | 1.55 | 1.25 | 1.93 |
|  |  | C | 7 | Mouse | Prkdc scid | SU-258 | overexpressed, level unknown | No | No | 5 | 5 | Antibody | IPeritoneal | Volume & Survival | 2.66 | 2.29 | 3.09 |
| Kramer-Marek, G.([Kramer-Marek et al., 2012](#_ENREF_41)) | 2012 |  | 5 | Mouse | athymic nude | BT-474 | 3+ | No | Yes | 18 | 24 | Vehicle | IPeritoneal | Volume | 2.18 | 1.90 | 2.51 |
| Kute, T. E.([Kute et al., 2009](#_ENREF_42)) | 2009 | A | 7 | Mouse | athymic nude | BT-474 TR | overexpressed, level unknown | Yes | Yes | 5 | 5 | Untreated | IPeritoneal | Volume | 6.02 | 4.76 | 7.60 |
|  |  | B | 7 | Mouse | athymic nude | BT-474 | overexpressed, level unknown | No | Yes | 5 | 5 | Untreated | IPeritoneal | Volume | 6.48 | 3.60 | 11.64 |
| Le, X. F.([Le et al., 2008](#_ENREF_44)) | 2008 | A | 4 | Mouse | nu/nu | BT-474 | 3+ | No | No | 5 | 5 | Antibody | IPeritoneal | Volume | 1.72 | 1.20 | 2.46 |
|  |  | B | 4 | Mouse | nu/nu | BT-474 | 3+ | No | No | 5 | 5 | Antibody | IPeritoneal | Volume | 2.94 | 2.07 | 4.16 |
| Lee, S.([Lee et al., 2002](#_ENREF_45)) | 2002 |  | 3 | Mouse | ICR SCID | 435.eB | overexpressed, level unknown | No | No | 9 | 9 | Antibody | IPeritoneal | Volume | 1.28 | 1.07 | 1.54 |
| Lee-Hoeflich, S. T.([Lee-Hoeflich et al., 2008](#_ENREF_46)) | 2008 | A | 6 | Mouse | beige nude XID | BT474M1 | 3+ | No | Yes | 3 | 3 | Vehicle | IPeritoneal | Volume | 0.79 | 0.61 | 1.04 |
|  |  | B | 6 | Mouse | beige nude XID | BT474M1 | 3+ | No | Yes | 3 | 3 | Vehicle | IPeritoneal | Volume | 17.49 | 13.71 | 22.31 |
|  |  | C | 6 | Mouse | beige nude XID | MDA-MB-175 | 1+ | No | No | 3 | 3 | Vehicle | IPeritoneal | Volume | 19.32 | 14.98 | 24.91 |
| Lewis Phillips, G. D.([Lewis Phillips et al., 2008](#_ENREF_47)) | 2008 | A | 9 | Mouse | nu/nu | MMTV | 3+ | Yes | No | 8 | 8 | Vehicle | IPeritoneal | Volume | 1.15 | 1.06 | 1.25 |
|  |  | B | 9 | Mouse | beige nude XID | BT-474EEI | 3+ | Yes | No | 10 | 10 | Vehicle | IVenous | Volume | 1.39 | 1.30 | 1.48 |
|  |  | C | 9 | Mouse | SCID beige | KPL-4 | 3+ | Yes | No | 8 | 8 | Vehicle | IPeritoneal | Volume | 7.35 | 6.86 | 7.86 |
| Liang, K.([Liang et al., 2010](#_ENREF_48)) | 2010 | A | 6 | Mouse | Swiss nude nu/nu | MCF7-HER18/Fluc-GFP | 1+ | No | No | 10 | 10 | Vehicle | IPeritoneal | Volume | 8.50 | 7.39 | 9.77 |
|  |  | B | 6 | Mouse | ICR SCID | MDA453beta | +- | No | No | 5 | 5 | Vehicle | IPeritoneal | Volume | 92.13 | 82.63 | 102.72 |
| Liu, X.([Liu et al., 2006](#_ENREF_49)) | 2006 |  | 5 | Mouse | athymic (CD-1 nu/nu) | BT-474 | 3+ | No | No | 7 | 7 | Vehicle | IPeritoneal | Volume | 1.27 | 1.03 | 1.56 |
| Lu, C. H.([Lu et al., 2007](#_ENREF_50)) | 2007 |  | 3 | Mouse | SCID | BT-474M1 | overexpressed, level unknown | No | No | 7 | 7 | Vehicle | Unknown | Volume | 1.29 | 0.94 | 1.77 |
| Magnifico, A.([Magnifico et al., 2009](#_ENREF_51)) | 2009 | A | 6 | Mouse | nude | MCF-7 HER | 1+ | No | Yes | 6 | 6 | Untreated | IPeritoneal | Volume | 1.26 | 1.23 | 1.30 |
|  |  | B | 6 | Mouse | nude | ZR75-1 | 1+ | No | Yes | 6 | 6 | Untreated | IPeritoneal | Volume | 2.44 | 2.32 | 2.57 |
|  |  | C | 6 | Mouse | nude | MDA-MB-361 | 3+ | No | No | 6 | 6 | Untreated | IPeritoneal | Volume | 4.79 | 4.01 | 5.71 |
| Mason, J. K.([Mason et al., 2013](#_ENREF_52)) | 2013 |  | 6 | Mouse | BALB/c nu/nu | BT-474 | 3+ | No | Yes | 16 | 16 | Vehicle | IPeritoneal | Survival |  |  |  |
| McKenzie, T.([McKenzie et al., 2004](#_ENREF_53)) | 2004 |  | 4 | Mouse | nude | MCF-7-Her-18 | overexpressed, level unknown | No | Yes | 3 | 3 | Vehicle | IPeritoneal | Volume | 2.18 | 1.89 | 2.51 |
| McLarty, K.([McLarty et al., 2009](#_ENREF_54)) | 2009 | A | 7 | Mouse | athymic CD1 nu/nu | MDA-MB-231 | 1+ | No | Yes | 7 | 7 | Vehicle | IPeritoneal | Volume | 1.05 | 0.80 | 1.38 |
|  |  | B | 7 | Mouse | athymic CD1 nu/nu | MDA-MB-361 | 3+ | No | Yes | 7 | 7 | Vehicle | IPeritoneal | Volume | 2.92 | 2.45 | 3.48 |
| Miller, T. W.([Miller et al., 2009](#_ENREF_55)) | 2009 | A | 7 | mouse | syngeneic wild-type FVB | MMTV | overexpressed, level unknown | No | No | 9 | 10 | Vehicle | IPeritoneal | Volume | 1.60 | 1.31 | 1.95 |
|  |  | B | 7 | mouse | syngeneic wild-type FVB | MMTV | overexpressed, level unknown | No | No | 10 | 10 | Vehicle | IPeritoneal | Volume | 3.61 | 2.69 | 4.86 |
|  |  | C | 7 | mouse | syngeneic wild-type FVB | MMTV | overexpressed, level unknown | No | No | 7 | 8 | Vehicle | IPeritoneal | Volume | 16.60 | 10.93 | 25.21 |
|  |  | D | 7 | mouse | syngeneic wild-type FVB | MMTV | overexpressed, level unknown | No | No | 15 | 10 | Vehicle | IPeritoneal | Volume | 30.19 | 17.99 | 50.68 |
|  |  | E | 7 | mouse | syngeneic wild-type FVB | MMTV | overexpressed, level unknown | No | No | 10 | 11 | Vehicle | IPeritoneal | Volume | 36.71 | 22.24 | 60.59 |
| Moulder, S. L.([Moulder et al., 2001](#_ENREF_56)) | 2001 |  | 5 | Mouse | Balb/C athymic nude | BT-474 | 3+ | No | Yes | 7 | 8 | Vehicle | IPeritoneal | Volume | 9.14 | 8.58 | 9.73 |
| O'Donovan, N.([O'Donovan et al., 2011](#_ENREF_57)) | 2011 |  | 6 | Mouse | Balb/C athymic nude | BT-474 -GFP | 3+ | No | Yes | 6 | 6 | Vehicle | IPeritoneal | Volume | 24752.98 | 7053.75 | 86862.96 |
| Oliveras-Ferraros, C.([Oliveras-Ferraros et al., 2012](#_ENREF_58)) | 2012 | A | 7 | Mouse | athymic nude | SLUG/SNAIL2 KD-JIMT1 | 1+ | No | No | 5 | 5 | Vehicle | IPeritoneal | Volume | 1.22 | 1.11 | 1.33 |
|  |  | B | 7 | Mouse | athymic nude | JIMT-1 | 1+ | Yes | No | 5 | 5 | Vehicle | IPeritoneal | Volume | 4.06 | 3.38 | 4.89 |
| Ono, N.([Ono et al., 2012](#_ENREF_59)) | 2012 |  | 7 | Mouse | athymic nude (BALB⁄c nu⁄nu) | BT-474 | overexpressed, level unknown | No | No | 4 | 4 | Vehicle | IPeritoneal | Volume | 2.28 | 1.86 | 2.79 |
| Rasaneh, S.([Rasaneh et al., 2012](#_ENREF_60)) | 2012 |  | 5 | Mouse | BALB/c | murine mammary carcinoma cells | +- | No | No | 10 | 10 | Vehicle | IVenous | Volume | 1.12 | 1.01 | 1.23 |
| Reyzer, M. L.([Reyzer et al., 2004](#_ENREF_61)) | 2004 | A | 7 | Mouse | FVB transgenic | MMTV | overexpressed, level unknown | No | No | 10 | 10 | Untreated | IPeritoneal | Volume | 0.91 | 0.76 | 1.10 |
|  |  | B | 7 | Mouse | FVB transgenic | MMTV | overexpressed, level unknown | No | No | 3 | 3 | Untreated | IPeritoneal | Volume | 150.69 | 89.68 | 253.21 |
| Ritter, C. A.([Ritter et al., 2004](#_ENREF_62)) | 2004 |  | 4 | Mouse | Balb/C athymic nude | BT-474 TR | overexpressed, level unknown | Yes | Yes | 8 | 8 | Vehicle | IPeritoneal | Volume | 0.81 | 0.70 | 0.94 |
| Ritter, C. A.([Ritter et al., 2007](#_ENREF_63)) | 2007 |  | 5 | Mouse | Balb/C athymic nude | BT-474 TR | 3+ | Yes | Yes | 8 | 8 | Untreated | IPeritoneal | Volume | 0.81 | 0.70 | 0.94 |
| Rodrigues, L. M.([Rodrigues et al., 2004](#_ENREF_64)) | 2004 |  | 4 | Mouse | FVB/n | Unknown | 3+ | No | No | 11 | 4 | Vehicle | IPeritoneal | Volume | 1.51 | 1.35 | 1.69 |
| Sabnis, G.([Sabnis et al., 2009](#_ENREF_65)) | 2009 | A | 7 | Mouse | ovarectomized, BALB/c athymic nude | LTLT-Ca | 1+ | No | No | 10 | 10 | Untreated | IPeritoneal | Volume | 3.20 | 2.40 | 4.27 |
|  |  | B | 7 | Mouse | ovarectomized, BALB/c athymic nude | LTLT-Ca | 1+ | No | No | 10 | 10 | Untreated | IPeritoneal | Volume | 8.74 | 5.99 | 12.77 |
|  |  | C | 7 | Mouse | ovarectomized, BALB/c athymic nude | LTLT-Ca | 1+ | No | No | 10 | 10 | Untreated | IPeritoneal | Volume | 12.27 | 8.10 | 18.59 |
|  |  | D | 7 | Mouse | ovarectomized, BALB/c athymic nude | LTLT-Ca | 1+ | No | No | 10 | 10 | Untreated | IPeritoneal | Volume | 28.50 | 18.17 | 44.70 |
| Scaltriti, M.([Scaltriti et al., 2007](#_ENREF_66)) | 2007 | A | 6 | Mouse | BALB/c athymic (nu+/nu+) | MCF-7 expressing truncated p95HER2 | 3+ | Yes | Yes | 8 | 8 | Vehicle | IPeritoneal | Volume | 1.03 | 0.97 | 1.08 |
|  |  | B | 6 | Mouse | BALB/c athymic (nu+/nu+) | MCF-7 | 1+ | No | Yes | 8 | 8 | Vehicle | IPeritoneal | Volume | 2.26 | 2.16 | 2.37 |
| Scheuer, W.([Scheuer et al., 2009](#_ENREF_67)) | 2009 |  | 6 | Mouse | SCID beige | KPL-4 | overexpressed, level unknown | No | No | 10 | 10 | Vehicle | IPeritoneal | Volume | 1.64 | 1.36 | 1.99 |
| Scotti, M. L.([Scotti et al., 2008](#_ENREF_68)) | 2008 | A | 5 | Mouse | athymic nude (nu/nu) | BT-474 | 3+ | No | Yes | 3 | 3 | Vehicle | IPeritoneal | Volume | 2.02 | 1.54 | 2.66 |
|  |  | B | 5 | Mouse | athymic nude (nu/nu) | T-47D | +- | No | Yes | 3 | 3 | Vehicle | IPeritoneal | Volume | 2.94 | 2.12 | 4.09 |
| Seoane, S.([Seoane et al., 2010](#_ENREF_69)) | 2010 |  | 6 | Mouse | BALB/c nude | BT-474 | 3+ | No | Yes | 6 | 6 | Vehicle | IPeritoneal | Volume | 2.61 | 2.43 | 2.81 |
| Shen, G.([Shen et al., 2011](#_ENREF_70)) | 2011 |  | 8 | Mouse | BALB/c nude | BT-474 | 3+ | No | Yes | 3 | 3 | Vehicle | IVenous | Volume | 5.88 | 4.59 | 7.53 |
| Smith, T. A.([Smith et al., 2013](#_ENREF_71)) | 2013 |  | 6 | Mouse | SCID | MDA-MB-453 | overexpressed, level unknown | No | No | 7 | 7 | Vehicle | IPeritoneal | Volume | 3.09 | 2.46 | 3.86 |
| Spiridon, C. I.([Spiridon et al., 2002](#_ENREF_72)) | 2002 |  | 4 | Mouse | preirradiated SCID | BT-474 | overexpressed, level unknown | No | No | 5 | 5 | Vehicle | IPeritoneal | Volume & Survival | 3.37 | 2.78 | 4.09 |
| Wang, C. X.([Wang et al., 2005](#_ENREF_73)) | 2005 |  | 4 | Mouse | ovarectomized, BALB/c athymic nude | BT-474 | 1+ | No | Yes | 5 | 5 | Vehicle | IPeritoneal | Volume | 5.68 | 4.05 | 7.98 |
| Wang, L. H.([Wang et al., 2007](#_ENREF_74)) | 2007 | A | 6 | Mouse | FVB/N-TGN (CMMTVneu) | MMTV | overexpressed, level unknown | No | No | 10 | 10 | Vehicle | IPeritoneal | Volume | 1.29 | 1.01 | 1.65 |
|  |  | B | 6 | Mouse | BALB/C athymic | MCF-7 I4 | overexpressed, level unknown | No | No | 5 | 5 | Vehicle | Unknown | Volume | 1.61 | 1.35 | 1.91 |
| Wang, S.([Wang et al., 2012](#_ENREF_75)) | 2012 |  | 7 | Mouse | BALB/c nude | BT-474 | overexpressed, level unknown | No | Yes | 10 | 10 | Antibody | IVenous | Volume & Survival | 1.91 | 1.83 | 1.99 |
| Warburton, C.([Warburton et al., 2004](#_ENREF_76)) | 2004 | A | 5 | Mouse | SCID Rag2-M | LCC6 | +- | No | Yes | 4 | 4 | Vehicle | IPeritoneal | Volume | 1.74 | 1.20 | 2.54 |
|  |  | B | 5 | Mouse | SCID Rag2-M | LCC6 | +- | No | Yes | 4 | 4 | Vehicle | IPeritoneal | Volume | 2.26 | 1.91 | 2.66 |
|  |  | C | 5 | Mouse | SCID Rag2-M | LCC6 | +- | No | Yes | 4 | 4 | Vehicle | IPeritoneal | Volume | 2.30 | 1.67 | 3.16 |
|  |  | D | 5 | Mouse | SCID Rag2-M | LCC6 | +- | No | Yes | 4 | 4 | Vehicle | IPeritoneal | Volume | 3.05 | 2.35 | 3.97 |
|  |  | E | 5 | Mouse | SCID Rag2-M | MCF-7 HER | 1+ | No | Yes | 10 | 10 | Vehicle | IPeritoneal | Volume | 3.24 | 2.46 | 4.26 |
|  |  | F | 5 | Mouse | SCID Rag2-M | LCC6 | +- | No | Yes | 4 | 4 | Vehicle | IPeritoneal | Volume | 7.62 | 5.43 | 10.69 |
| Waterhouse, D. N.([Waterhouse et al., 2005](#_ENREF_77)) | 2005 | A | 6 | Mouse | Rag2-M | MCF-7 HER2 | overexpressed, level unknown | No | Yes | 5 | 5 | Vehicle | IPeritoneal | Volume | 1.58 | 0.93 | 2.68 |
|  |  | B | 6 | Mouse | Rag2-M | MCF-7 HER2 | overexpressed, level unknown | No | Yes | 5 | 5 | Vehicle | IPeritoneal | Volume | 9.73 | 5.12 | 18.48 |
|  |  | C | 6 | Mouse | Rag2-M | MCF-7 HER2 | overexpressed, level unknown | No | Yes | 5 | 5 | Vehicle | IPeritoneal | Volume | 11.19 | 5.44 | 22.99 |
|  |  | D | 6 | Mouse | Rag2-M | MCF-7 HER2 | overexpressed, level unknown | No | Yes | 5 | 5 | Vehicle | IPeritoneal | Volume | 10896.22 | 3081.42 | 38530.16 |
| Wen, X. F.([Wen et al., 2006](#_ENREF_78)) | 2006 |  | 6 | Mouse | BALB/c athymic nu/nu | BT-474 | 3+ | No | Yes | 7 | 7 | Antibody | IPeritoneal | Volume | 1.84 | 1.61 | 2.11 |
| Zhang, N.([Zhang et al., 2011](#_ENREF_79)) | 2011 | A | 5 | Mouse | Immunodeficient  nu/nu | BT-474M1 | overexpressed, level unknown | No | No | 10 | 10 | Vehicle | Unknown | Volume | 0.93 | 0.81 | 1.06 |
|  |  | B | 5 | Mouse | Immunodeficient  nu/nu | BT-474M1 | overexpressed, level unknown | No | No | 10 | 10 | Vehicle | Unknown | Volume | 1.83 | 1.59 | 2.10 |
|  |  | C | 5 | Mouse | Immunodeficient  nu/nu | BT-474M1 | overexpressed, level unknown | No | No | 10 | 10 | Vehicle | Unknown | Volume | 15.85 | 13.26 | 18.94 |
| Zhao, Y.([Zhao et al., 2011](#_ENREF_80)) | 2011 | A | 6 | Mouse | athymic nude | BT-474 TR | overexpressed, level unknown | Yes | Yes | 8 | 8 | Vehicle | IPeritoneal | Volume | 1.17 | 1.07 | 1.29 |
|  |  | B | 6 | Mouse | athymic nude | BT-474 | overexpressed, level unknown | No | Yes | 8 | 8 | Vehicle | IPeritoneal | Volume | 3.03 | 2.15 | 4.28 |
| Zhu, Y.([Zhu et al., 2012](#_ENREF_81)) | 2012 |  | 7 | Mouse | BALB/c nude | BT-474 | overexpressed, level unknown | No | No | 5 | 5 | Vehicle | Unknown | Volume | 1.35 | 1.23 | 1.48 |
| Zhuang, G.([Zhuang et al., 2010](#_ENREF_82)) | 2010 |  | 5 | Mouse | athymic nude | BT-474 TR | 3+ | Yes | Yes | 10 | 10 | Antibody | IPeritoneal | Volume | 1.01 | 0.84 | 1.21 |

ABBAS, N., HEYERDAHL, H., BRULAND, O. S., BORREBAEK, J., NESLAND, J. & DAHLE, J. 2011. Experimental (alpha)-particle radioimmunotherapy of breast cancer using 227Th-labeled p-benzyl-DOTA-trastuzumab. *EJNMMI Research,* 1**,** 1-12.

AGUS, D. B., AKITA, R. W., FOX, W. D., LEWIS, G. D., HIGGINS, B., PISACANE, P. I., LOFGREN, J. A., TINDELL, C., EVANS, D. P., MAIESE, K., SCHER, H. I. & SLIWKOWSKI, M. X. 2002. Targeting ligand-activated ErbB2 signaling inhibits breast and prostate tumor growth. *Cancer Cell,* 2**,** 127-37.

ANIDO, J., SCALTRITI, M., BECH SERRA, J. J., SANTIAGO JOSEFAT, B., TODO, F. R., BASELGA, J. & ARRIBAS, J. 2006. Biosynthesis of tumorigenic HER2 C-terminal fragments by alternative initiation of translation. *EMBO J,* 25**,** 3234-44.

ARNAL-ESTAPE, A., TARRAGONA, M., MORALES, M., GUIU, M., NADAL, C., MASSAGUE, J. & GOMIS, R. R. 2010. HER2 silences tumor suppression in breast cancer cells by switching expression of C/EBPss isoforms. *Cancer Res,* 70**,** 9927-36.

BAROK, M., BALAZS, M., NAGY, P., RAKOSY, Z., TRESZL, A., TOTH, E., JUHASZ, I., PARK, J. W., ISOLA, J., VEREB, G. & SZOLLOSI, J. 2008. Trastuzumab decreases the number of circulating and disseminated tumor cells despite trastuzumab resistance of the primary tumor. *Cancer Lett,* 260**,** 198-208.

BAROK, M., ISOLA, J., PALYI-KREKK, Z., NAGY, P., JUHASZ, I., VEREB, G., KAURANIEMI, P., KAPANEN, A., TANNER, M., VEREB, G. & SZOLLOSI, J. 2007. Trastuzumab causes antibody-dependent cellular cytotoxicity-mediated growth inhibition of submacroscopic JIMT-1 breast cancer xenografts despite intrinsic drug resistance. *Mol Cancer Ther,* 6**,** 2065-72.

BASELGA, J., NORTON, L., ALBANELL, J., KIM, Y. M. & MENDELSOHN, J. 1998. Recombinant humanized anti-HER2 antibody (herceptin(TM)) enhances the antitumor activity of paclitaxel and doxorubicin against HER2/neu overexpressing human breast cancer xenografts. *Cancer Research,* 58**,** 2825-2831.

BEYER, I., VAN RENSBURG, R., STRAUSS, R., LI, Z., WANG, H., PERSSON, J., YUMUL, R., FENG, Q., SONG, H., BARTEK, J., FENDER, P. & LIEBER, A. 2011. Epithelial junction opener JO-1 improves monoclonal antibody therapy of cancer. *Cancer Res,* 71**,** 7080-90.

BOCANGEL, D., ZHENG, M., MHASHILKAR, A., LIU, Y., RAMESH, R., HUNT, K. K. & CHADA, S. 2006. Combinatorial synergy induced by adenoviral-mediated mda-7 and Herceptin in Her-2+ breast cancer cells. *Cancer Gene Ther,* 13**,** 958-68.

BRODIE, A., SABNIS, G. & MACEDO, L. 2007. Xenograft models for aromatase inhibitor studies. *J Steroid Biochem Mol Biol,* 106**,** 119-24.

CAPIETTO, A. H., MARTINET, L. & FOURNIE, J. J. 2011. Stimulated (gamma)(delta) T cells increase the in vivo efficacy of trastuzumab in HER-2+ breast cancer. *Journal of Immunology,* 187**,** 1031-1038.

CHAKRABARTY, A., BHOLA, N. E., SUTTON, C., GHOSH, R., KUBA, M. G., DAVE, B., CHANG, J. C. & ARTEAGA, C. L. 2013. Trastuzumab-resistant cells rely on a HER2-PI3K-FoxO-survivin axis and are sensitive to PI3K inhibitors. *Cancer Res,* 73**,** 1190-200.

CHAKRABARTY, A., SANCHEZ, V., KUBA, M. G., RINEHART, C. & ARTEAGA, C. L. 2012. Feedback upregulation of HER3 (ErbB3) expression and activity attenuates antitumor effect of PI3K inhibitors. *Proc Natl Acad Sci U S A,* 109**,** 2718-23.

CHAN, C. H., LI, C. F., YANG, W. L., GAO, Y., LEE, S. W., FENG, Z., HUANG, H. Y., TSAI, K. K., FLORES, L. G., SHAO, Y., HAZLE, J. D., YU, D., WEI, W., SARBASSOV, D., HUNG, M. C., NAKAYAMA, K. I. & LIN, H. K. 2012. The Skp2-SCF E3 ligase regulates Akt ubiquitination, glycolysis, herceptin sensitivity, and tumorigenesis. *Cell,* 149**,** 1098-111.

CHEUNG, N. K., MODAK, S., VICKERS, A. & KNUCKLES, B. 2002. Orally administered beta-glucans enhance anti-tumor effects of monoclonal antibodies. *Cancer Immunol Immunother,* 51**,** 557-64.

CHING, C. L., CHESEBROUGH, J., COFFMAN, K. T., FAZENBAKER, C. A., GOOYA, J., WENG, D., COATS, S., JACKSON, D., JALLAL, B. & CHANG, Y. 2009. Antitumor efficacy of IPI-504, a selective heat shock protein 90 inhibitor against human epidermal growth factor receptor 2-positive human xenograft models as a single agent and in combination with trastuzumab or lapatinib. *Molecular Cancer Therapeutics,* 8**,** 2131-2141.

CHIU, G. N., EDWARDS, L. A., KAPANEN, A. I., MALINEN, M. M., DRAGOWSKA, W. H., WARBURTON, C., CHIKH, G. G., FANG, K. Y., TAN, S., SY, J., TUCKER, C., WATERHOUSE, D. N., KLASA, R. & BALLY, M. B. 2007. Modulation of cancer cell survival pathways using multivalent liposomal therapeutic antibody constructs. *Mol Cancer Ther,* 6**,** 844-55.

CHUMSRI, S., SABNIS, G. J., HOWES, T. & BRODIE, A. M. 2011. Aromatase inhibitors and xenograft studies. *Steroids,* 76**,** 730-5.

COLBERN, G. T., HILLER, A. J., MUSTERER, R. S., WORKING, P. K. & HENDERSON, I. C. 1999. Antitumor activity of Herceptin(registered trademark) in combination with STEALTH(registered trademark) liposomal cisplatin or nonliposomal cisplatin in a HER2 positive human breast cancer model. *Journal of Inorganic Biochemistry,* 77**,** 117-120.

COSTANTINI, D. L., MCLARTY, K., LEE, H., DONE, S. J., VALLIS, K. A. & REILLY, R. M. 2010. Antitumor effects and normal-tissue toxicity of111In-nuclear localization sequence-trastuzumab in Athymic mice bearing HER-positive human breast cancer xenografts. *Journal of Nuclear Medicine,* 51**,** 1084-1091.

DAMIANO, V., GAROFALO, S., ROSA, R., BIANCO, R., CAPUTO, R., GELARDI, T., MEROLA, G., RACIOPPI, L., GARBI, C., KANDIMALLA, E. R., AGRAWAL, S. & TORTORA, G. 2009. A novel toll-like receptor 9 agonist cooperates with trastuzumab in trastuzumab-resistant breast tumors through multiple mechanisms of action. *Clinical Cancer Research,* 15**,** 6921-6930.

FOY, K. C., MILLER, M. J., MOLDOVAN, N., BOZANOVIC, T., CARSON III, W. E. & KAUMAYA, P. T. 2012. Immunotherapy with HER-2 and VEGF peptide mimics plus metronomic paclitaxel causes superior antineoplastic effects in transplantable and transgenic mouse models of human breast cancer. *Oncoimmunology,* 1**,** 1004-1016.

FRANCIA, G., MAN, S., LEE, C. J., LEE, C. R., XU, P., MOSSOBA, M. E., EMMENEGGER, U., MEDIN, J. A. & KERBEL, R. S. 2009. Comparative impact of trastuzumab and cyclophosphamide on HER-2-positive human breast cancer xenografts. *Clin Cancer Res,* 15**,** 6358-66.

FUJIMOTO-OUCHI, K., SEKIGUCHI, F. & TANAKA, Y. 2002. Antitumor activity of combinations of anti-HER-2 antibody trastuzumab and oral fluoropyrimidines capecitabine/5'-dFUrd in human breast cancer models. *Cancer Chemother Pharmacol,* 49**,** 211-6.

FUJIMOTO-OUCHI, K., SEKIGUCHI, F., YAMAMOTO, K., SHIRANE, M., YAMASHITA, Y. & MORI, K. 2010. Preclinical study of prolonged administration of trastuzumab as combination therapy after disease progression during trastuzumab monotherapy. *Cancer Chemotherapy and Pharmacology,* 66**,** 269-276.

GARRETT, J. T., SUTTON, C. R., COOK, R. S. & ARTEAGA, C. L. 2012. Dual blockade of HER2 in HER2-overexpressing tumor cells does not eliminate HER3 function completely: Clinical implications. *Cancer Research,* 72.

GEE, M. S., UPADHYAY, R., BERGQUIST, H., ALENCAR, H., REYNOLDS, F., MARICEVICH, M., WEISSLEDER, R., JOSEPHSON, L. & MAHMOOD, U. 2008. Human breast cancer tumor models: molecular imaging of drug susceptibility and dosing during HER2/neu-targeted therapy. *Radiology,* 248**,** 925-35.

GEE, M. S., UPADHYAY, R., BERGQUIST, H., WEISSLEDER, R., JOSEPHSON, L. & MAHMOOD, U. 2007. Multiparameter noninvasive assessment of treatment susceptibility, drug target inhibition and tumor response guides cancer treatment. *Int J Cancer,* 121**,** 2492-500.

GIJSEN, M., KING, P., PERERA, T., PARKER, P. J., HARRIS, A. L., LARIJANI, B. & KONG, A. 2010. HER2 phosphorylation is maintained by a PKB negative feedback loop in response to anti-HER2 herceptin in breast cancer. *PLoS biology,* 8**,** e1000563.

HAN, H. & DAVIS, M. E. 2013. Single-Antibody, Targeted Nanoparticle Delivery of Camptothecin. *Mol Pharm*.

HEYERDAHL, H., ABBAS, N., BREVIK, E. M., MOLLATT, C. & DAHLE, J. 2012. Fractionated therapy of HER2-expressing breast and ovarian cancer xenografts in mice with targeted alpha emitting 227Th-DOTA-p-benzyl-trastuzumab. *PLoS One,* 7**,** e42345.

INOUE, S., DING, H., PORTILLA-ARIAS, J., HU, J., KONDA, B., FUJITA, M., ESPINOZA, A., SUHANE, S., RILEY, M., GATES, M., PATIL, R., PENICHET, M. L., LJUBIMOV, A. V., BLACK, K. L., HOLLER, E. & LJUBIMOVA, J. Y. 2011. Polymalic acid-based nanobiopolymer provides efficient systemic breast cancer treatment by inhibiting both HER2/neu receptor synthesis and activity. *Cancer Research,* 71**,** 1454-1464.

ITHIMAKIN, S., DAY, K. C., MALIK, F., ZEN, Q., DAWSEY, S. J., BERSANO-BEGEY, T. F., QURAISHI, A. A., IGNATOSKI, K. W., DAIGNAULT, S., DAVIS, A., HALL, C. L., PALANISAMY, N., HEATH, A. N., TAWAKKOL, N., LUTHER, T. K., CLOUTHIER, S. G., CHADWICK, W. A., DAY, M. L., KLEER, C. G., THOMAS, D. G., HAYES, D. F., KORKAYA, H. & WICHA, M. S. 2013. HER2 drives luminal breast cancer stem cells in the absence of HER2 amplification: implications for efficacy of adjuvant trastuzumab. *Cancer Res,* 73**,** 1635-46.

JEROME, L., ALAMI, N., BELANGER, S., PAGE, V., YU, Q., PATERSON, J., SHIRY, L., PEGRAM, M. & LEYLAND-JONES, B. 2006. Recombinant human insulin-like growth factor binding protein 3 inhibits growth of human epidermal growth factor receptor-2-overexpressing breast tumors and potentiates herceptin activity in vivo. *Cancer Res,* 66**,** 7245-52.

JUMBE, N. L., XIN, Y., LEIPOLD, D. D., CROCKER, L., DUGGER, D., MAI, E., SLIWKOWSKI, M. X., FIELDER, P. J. & TIBBITTS, J. 2010. Modeling the efficacy of trastuzumab-DM1, an antibody drug conjugate, in mice. *J Pharmacokinet Pharmacodyn,* 37**,** 221-42.

JUNTTILA, T. T., AKITA, R. W., PARSONS, K., FIELDS, C., LEWIS PHILLIPS, G. D., FRIEDMAN, L. S., SAMPATH, D. & SLIWKOWSKI, M. X. 2009. Ligand-Independent HER2/HER3/PI3K Complex Is Disrupted by Trastuzumab and Is Effectively Inhibited by the PI3K Inhibitor GDC-0941. *Cancer Cell,* 15**,** 429-440.

JUNTTILA, T. T., LI, G., PARSONS, K., PHILLIPS, G. L. & SLIWKOWSKI, M. X. 2011. Trastuzumab-DM1 (T-DM1) retains all the mechanisms of action of trastuzumab and efficiently inhibits growth of lapatinib insensitive breast cancer. *Breast Cancer Res Treat,* 128**,** 347-56.

JUNTTILA, T. T., PARSONS, K., OLSSON, C., LU, Y., XIN, Y., THERIAULT, J., CROCKER, L., PABONAN, O., BAGINSKI, T., MENG, G., TOTPAL, K., KELLEY, R. F. & SLIWKOWSKI, M. X. 2010. Superior in vivo efficacy of afucosylated trastuzumab in the treatment of HER2-amplified breast cancer. *Cancer Res,* 70**,** 4481-9.

KLOS, K. S., ZHOU, X., LEE, S., ZHANG, L., YANG, W., NAGATA, Y. & YU, D. 2003. Combined trastuzumab and paclitaxel treatment better inhibits ErbB-2-mediated angiogenesis in breast carcinoma through a more effective inhibition of Akt than either treatment alone. *Cancer,* 98**,** 1377-1385.

KOHRT, H. E., HOUOT, R., WEISKOPF, K., GOLDSTEIN, M. J., SCHEEREN, F., CZERWINSKI, D., COLEVAS, A. D., WENG, W. K., CLARKE, M. F., CARLSON, R. W., STOCKDALE, F. E., MOLLICK, J. A., CHEN, L. & LEVY, R. 2012. Stimulation of natural killer cells with a CD137-specific antibody enhances trastuzumab efficacy in xenotransplant models of breast cancer. *J Clin Invest,* 122**,** 1066-75.

KRAMER-MAREK, G., GIJSEN, M., KIESEWETTER, D. O., BENNETT, R., ROXANIS, I., ZIELINSKI, R., KONG, A. & CAPALA, J. 2012. Potential of PET to predict the response to trastuzumab treatment in an ErbB2-positive human xenograft tumor model. *J Nucl Med,* 53**,** 629-37.

KUTE, T. E., SAVAGE, L., STEHLE, J. R., JR., KIM-SHAPIRO, J. W., BLANKS, M. J., WOOD, J. & VAUGHN, J. P. 2009. Breast tumor cells isolated from in vitro resistance to trastuzumab remain sensitive to trastuzumab anti-tumor effects in vivo and to ADCC killing. *Cancer Immunol Immunother,* 58**,** 1887-96.

LAI, H. W., CHEN, D. R., CHIEN, S. Y., KUO, S. J., TSENG, L. M., LIN, H. Y. & CHI, C. W. 2012. The potential utility of curcumin in the treatment of HER-2-overexpressed breast cancer: An in vitro and in vivo comparison study with herceptin. *Evidence-based Complementary and Alternative Medicine,* 2012.

LE, X. F., MAO, W., LU, C., THORNTON, A., HEYMACH, J. V., SOOD, A. K. & BAST JR, R. C. 2008. Specific blockade of VEGF and HER2 pathways results in greater growth inhibition of breast cancer xenografts that overexpress HER2. *Cell Cycle,* 7**,** 3747-3758.

LEE, S., YANG, W., LAN, K. H., SELLAPPAN, S., KLOS, K., HORTOBAGYI, G., HUNG, M. C. & YU, D. 2002. Enhanced sensitization to taxol-induced apoptosis by herceptin pretreatment in ErbB2-overexpressing breast cancer cells. *Cancer Res,* 62**,** 5703-10.

LEE-HOEFLICH, S. T., CROCKER, L., YAO, E., PHAM, T., MUNROE, X., HOEFLICH, K. P., SLIWKOWSKI, M. X. & STERN, H. M. 2008. A central role for HER3 in HER2-amplified breast cancer: implications for targeted therapy. *Cancer Res,* 68**,** 5878-87.

LEWIS PHILLIPS, G. D., LI, G., DUGGER, D. L., CROCKER, L. M., PARSONS, K. L., MAI, E., BLATTLER, W. A., LAMBERT, J. M., CHARI, R. V., LUTZ, R. J., WONG, W. L., JACOBSON, F. S., KOEPPEN, H., SCHWALL, R. H., KENKARE-MITRA, S. R., SPENCER, S. D. & SLIWKOWSKI, M. X. 2008. Targeting HER2-positive breast cancer with trastuzumab-DM1, an antibody-cytotoxic drug conjugate. *Cancer Res,* 68**,** 9280-90.

LIANG, K., ESTEVA, F. J., ALBARRACIN, C., STEMKE-HALE, K., LU, Y., BIANCHINI, G., YANG, C. Y., LI, Y., LI, X., CHEN, C. T., MILLS, G. B., HORTOBAGYI, G. N., MENDELSOHN, J., HUNG, M. C. & FAN, Z. 2010. Recombinant human erythropoietin antagonizes trastuzumab treatment of breast cancer cells via Jak2-mediated Src activation and PTEN inactivation. *Cancer Cell,* 18**,** 423-35.

LIU, X., FRIDMAN, J. S., WANG, Q., CAULDER, E., YANG, G., COVINGTON, M., LIU, C., MARANDO, C., ZHUO, J., LI, Y., YAO, W., VADDI, K., NEWTON, R. C., SCHERLE, P. A. & FRIEDMAN, S. M. 2006. Selective inhibition of ADAM metalloproteases blocks HER-2 extracellular domain (ECD) cleavage and potentiates the anti-tumor effects of trastuzumab. *Cancer Biol Ther,* 5**,** 648-56.

LU, C. H., WYSZOMIERSKI, S. L., TSENG, L. M., SUN, M. H., LAN, K. H., NEAL, C. L., MILLS, G. B., HORTOBAGYI, G. N., ESTEVA, F. J. & YU, D. 2007. Preclinical testing of clinically applicable strategies for overcoming trastuzumab resistance caused by PTEN deficiency. *Clin Cancer Res,* 13**,** 5883-8.

MAGNIFICO, A., ALBANO, L., CAMPANER, S., DELIA, D., CASTIGLIONI, F., GASPARINI, P., SOZZI, G., FONTANELLA, E., MENARD, S. & TAGLIABUE, E. 2009. Tumor-initiating cells of HER2-positive carcinoma cell lines express the highest oncoprotein levels and are sensitive to trastuzumab. *Clin Cancer Res,* 15**,** 2010-21.

MASON, J. K., FU, M. H., CHEN, J., YU, Z. & THOMPSON, L. U. 2013. Dietary flaxseed-trastuzumab interactive effects on the growth of HER2-overexpressing human breast tumors (BT-474). *Nutr Cancer,* 65**,** 451-9.

MCKENZIE, T., LIU, Y., FANALE, M., SWISHER, S. G., CHADA, S. & HUNT, K. K. 2004. Combination therapy of Ad-mda7 and trastuzumab increases cell death in Her-2/neu-overexpressing breast cancer cells. *Surgery,* 136**,** 437-42.

MCLARTY, K., FASIH, A., SCOLLARD, D. A., DONE, S. J., VINES, D. C., GREEN, D. E., COSTANTINI, D. L. & REILLY, R. M. 2009. 18F-FDG small-animal PET/CT differentiates trastuzumab-responsive from unresponsive human breast cancer xenografts in athymic mice. *J Nucl Med,* 50**,** 1848-56.

MILLER, T. W., FORBES, J. T., SHAH, C., WYATT, S. K., MANNING, H. C., OLIVARES, M. G., SANCHEZ, V., DUGGER, T. C., DE MATOS GRANJA, N., NARASANNA, A., COOK, R. S., KENNEDY, J. P., LINDSLEY, C. W. & ARTEAGA, C. L. 2009. Inhibition of mammalian target of rapamycin is required for optimal antitumor effect of HER2 inhibitors against HER2-overexpressing cancer cells. *Clin Cancer Res,* 15**,** 7266-76.

MOULDER, S. L., YAKES, F. M., MUTHUSWAMY, S. K., BIANCO, R., SIMPSON, J. F. & ARTEAGA, C. L. 2001. Epidermal growth factor receptor (HER1) tyrosine kinase inhibitor ZD1839 (Iressa) inhibits HER2/neu (erbB2)-overexpressing breast cancer cells in vitro and in vivo. *Cancer Res,* 61**,** 8887-95.

O'DONOVAN, N., BYRNE, A. T., O'CONNOR, A. E., MCGEE, S., GALLAGHER, W. M. & CROWN, J. 2011. Synergistic interaction between trastuzumab and EGFR/HER-2 tyrosine kinase inhibitors in HER-2 positive breast cancer cells. *Investigational New Drugs,* 29**,** 752-759.

OLIVERAS-FERRAROS, C., COROMINAS-FAJA, B., CUFI, S., VAZQUEZ-MARTIN, A., MARTIN-CASTILLO, B., IGLESIAS, J. M., LOPEZ-BONET, E., MARTIN, A. G. & MENENDEZ, J. A. 2012. Epithelial-to-mesenchymal transition (EMT) confers primary resistance to trastuzumab (Herceptin). *Cell Cycle,* 11**,** 4020-32.

ONO, N., YAMAZAKI, T., NAKANISHI, Y., FUJII, T., SAKATA, K., TACHIBANA, Y., SUDA, A., HADA, K., MIURA, T., SATO, S., SAITOH, R., NAKANO, K., TSUKUDA, T., MIO, T., ISHII, N., KONDOH, O. & AOKI, Y. 2012. Preclinical antitumor activity of the novel heat shock protein 90 inhibitor CH5164840 against human epidermal growth factor receptor 2 (HER2)-overexpressing cancers. *Cancer Sci,* 103**,** 342-9.

RASANEH, S., RAJABI, H., AKHLAGHPOOR, S. & SHEYBANI, S. 2012. Radioimmunotherapy of mice bearing breast tumors with 177Lu- labeled trastuzumab. *Turkish Journal of Medical Sciences,* 42**,** 1292-1298.

REYZER, M. L., CALDWELL, R. L., DUGGER, T. C., FORBES, J. T., RITTER, C. A., GUIX, M., ARTEAGA, C. L. & CAPRIOLI, R. M. 2004. Early changes in protein expression detected by mass spectrometry predict tumor response to molecular therapeutics. *Cancer Res,* 64**,** 9093-100.

RITTER, C. A., BIANCO, R., DUGGER, T., FORBES, J., QU, S., RINEHART, C., KING, W. & ARTEAGA, C. L. 2004. Mechanisms of resistance development against trastuzumab (Herceptin) in an in vivo breast cancer model. *Int J Clin Pharmacol Ther,* 42**,** 642-3.

RITTER, C. A., PEREZ-TORRES, M., RINEHART, C., GUIX, M., DUGGER, T., ENGELMAN, J. A. & ARTEAGA, C. L. 2007. Human breast cancer cells selected for resistance to trastuzumab in vivo overexpress epidermal growth factor receptor and ErbB ligands and remain dependent on the ErbB receptor network. *Clin Cancer Res,* 13**,** 4909-19.

RODRIGUES, L. M., STUBBS, M., ROBINSON, S. P., NEWELL, B., MANSI, J. & GRIFFITHS, J. R. 2004. The C-neu mammary carcinoma in Oncomice; characterization and monitoring response to treatment with herceptin by magnetic resonance methods. *MAGMA,* 17**,** 260-70.

SABNIS, G., SCHAYOWITZ, A., GOLOUBEVA, O., MACEDO, L. & BRODIE, A. 2009. Trastuzumab reverses letrozole resistance and amplifies the sensitivity of breast cancer cells to estrogen. *Cancer Res,* 69**,** 1416-28.

SCALTRITI, M., ROJO, F., OCANA, A., ANIDO, J., GUZMAN, M., CORTES, J., DI COSIMO, S., MATIAS-GUIU, X., RAMON Y CAJAL, S., ARRIBAS, J. & BASELGA, J. 2007. Expression of p95HER2, a truncated form of the HER2 receptor, and response to anti-HER2 therapies in breast cancer. *J Natl Cancer Inst,* 99**,** 628-38.

SCHEUER, W., FRIESS, T., BURTSCHER, H., BOSSENMAIER, B., ENDL, J. & HASMANN, M. 2009. Strongly enhanced antitumor activity of trastuzumab and pertuzumab combination treatment on HER2-positive human xenograft tumor models. *Cancer Res,* 69**,** 9330-6.

SCOTTI, M. L., LANGENHEIM, J. F., TOMBLYN, S., SPRINGS, A. E. & CHEN, W. Y. 2008. Additive effects of a prolactin receptor antagonist, G129R, and herceptin on inhibition of HER2-overexpressing breast cancer cells. *Breast Cancer Res Treat,* 111**,** 241-50.

SEOANE, S., MONTERO, J. C., OCANA, A. & PANDIELLA, A. 2010. Effect of multikinase inhibitors on caspase-independent cell death and DNA damage in HER2-overexpressing breast cancer cells. *J Natl Cancer Inst,* 102**,** 1432-46.

SHEN, G., HUANG, H., ZHANG, A., ZHAO, T., HU, S., CHENG, L., LIU, J., XIAO, W., LING, B., WU, Q., SONG, L. & WEI, W. 2011. In vivo activity of novel anti-ErbB2 antibody chA21 alone and with Paclitaxel or Trastuzumab in breast and ovarian cancer xenograft models. *Cancer Immunol Immunother,* 60**,** 339-48.

SMITH, T. A., APPLEYARD, M. V., SHARP, S., FLEMING, I. N., MURRAY, K. & THOMPSON, A. M. 2013. Response to trastuzumab by HER2 expressing breast tumour xenografts is accompanied by decreased Hexokinase II, glut1 and [18F]-FDG incorporation and changes in 31P-NMR-detectable phosphomonoesters. *Cancer Chemother Pharmacol,* 71**,** 473-80.

SPIRIDON, C. I., GHETIE, M. A., UHR, J., MARCHES, R., LI, J. L., SHEN, G. L. & VITETTA, E. S. 2002. Targeting multiple Her-2 epitopes with monoclonal antibodies results in improved antigrowth activity of a human breast cancer cell line in vitro and in vivo. *Clin Cancer Res,* 8**,** 1720-30.

WANG, C. X., KOAY, D. C., EDWARDS, A., LU, Z., MOR, G., OCAL, I. T. & DIGIOVANNA, M. P. 2005. In vitro and in vivo effects of combination of Trastuzumab (Herceptin) and Tamoxifen in breast cancer. *Breast Cancer Res Treat,* 92**,** 251-63.

WANG, L. H., CHAN, J. L. & LI, W. 2007. Rapamycin together with herceptin significantly increased anti-tumor efficacy compared to either alone in ErbB2 over expressing breast cancer cells. *Int J Cancer,* 121**,** 157-64.

WANG, S., CHEN, C., MENG, Y., HU, S., ZHENG, L., SONG, J., ZHANG, D., LI, B. & GUO, Y. 2012. Effective suppression of breast tumor growth by an anti-EGFR/ErbB2 bispecific antibody. *Cancer Lett,* 325**,** 214-9.

WARBURTON, C., DRAGOWSKA, W. H., GELMON, K., CHIA, S., YAN, H., MASIN, D., DENYSSEVYCH, T., WALLIS, A. E. & BALLY, M. B. 2004. Treatment of HER-2/neu overexpressing breast cancer xenograft models with trastuzumab (Herceptin) and gefitinib (ZD1839): drug combination effects on tumor growth, HER-2/neu and epidermal growth factor receptor expression, and viable hypoxic cell fraction. *Clin Cancer Res,* 10**,** 2512-24.

WATERHOUSE, D. N., DENYSSEVYCH, T., HUDON, N., CHIA, S., GELMON, K. A. & BALLY, M. B. 2005. Trastuzumab and liposomal doxorubicin in the treatment of MCF-7 xenograft tumor-bearing mice: Combination does not affect drug serum levels. *Pharmaceutical Research,* 22**,** 915-922.

WEN, X. F., YANG, G., MAO, W., THORNTON, A., LIU, J., BAST, R. C., JR. & LE, X. F. 2006. HER2 signaling modulates the equilibrium between pro- and antiangiogenic factors via distinct pathways: implications for HER2-targeted antibody therapy. *Oncogene,* 25**,** 6986-96.

ZHANG, N., LIU, L., DUMITRU, C. D., CUMMINGS, N. R., CUKAN, M., JIANG, Y., LI, Y., LI, F., MITCHELL, T., MALLEM, M. R., OU, Y., PATEL, R. N., VO, K., WANG, H., BURNINA, I., CHOI, B. K., HUBER, H. E., STADHEIM, T. A. & ZHA, D. 2011. Glycoengineered Pichia produced anti-HER2 is comparable to trastuzumab in preclinical study. *MAbs,* 3**,** 289-98.

ZHAO, Y., LIU, H., LIU, Z., DING, Y., LEDOUX, S. P., WILSON, G. L., VOELLMY, R., LIN, Y., LIN, W., NAHTA, R., LIU, B., FODSTAD, O., CHEN, J., WU, Y., PRICE, J. E. & TAN, M. 2011. Overcoming trastuzumab resistance in breast cancer by targeting dysregulated glucose metabolism. *Cancer Res,* 71**,** 4585-97.

ZHU, Y., ZHANG, X., LIU, Y., ZHANG, S., LIU, J., MA, Y. & ZHANG, J. 2012. Antitumor effect of the mTOR inhibitor everolimus in combination with trastuzumab on human breast cancer stem cells in vitro and in vivo. *Tumour Biol,* 33**,** 1349-62.

ZHUANG, G., BRANTLEY-SIEDERS, D. M., VAUGHT, D., YU, J., XIE, L., WELLS, S., JACKSON, D., MURAOKA-COOK, R., ARTEAGA, C. & CHEN, J. 2010. Elevation of receptor tyrosine kinase EphA2 mediates resistance to trastuzumab therapy. *Cancer Res,* 70**,** 299-308.
