# Supplementary material for: The Efficacy of Trastuzumab in Animal Models of Breast Cancer: A Systematic Review and Meta-Analysis
Source: PLoS One. 2016 Jul 27;11(7):e0158240. doi: 10.1371/journal.pone.0158240 (PMC4963137; doi:10.1371/journal.pone.0158240)
Supplement: S3 Table — (DOCX) [file pone.0158240.s004.docx]

Supplementary Material: Study Quality Scores
1) Peer-reviewed publication
2) Randomized allocation of tumour-bearing mice into treatment and control groups
3) Blinded assessment of outcome
4) Sample size calculation performed
5) Statement of potential conflict of interests
6) Compliance with animal welfare policy
7) Explanation of rationale for disease model used, or more than one model assessed for comparison
8) Standardised number or volume of tumour cells implanted
9) Reported number of animals in which the tumour did not grow
10) Reporting and explanation of excluded animals
11) Presentation of evidence that trastuzumab acts directly against the tumour
12) Consistent implantation site

| Name | Year | 1 | 2 | 3 | 4 | 5 | 6 | 7 | 8 | 9 | 10 | 11 | 12 | Quality Score |
| --- | --- | --- | --- | --- | --- | --- | --- | --- | --- | --- | --- | --- | --- | --- |
| Abbas, N. | 2011 | + |  |  |  | + | + |  | + |  | + | + |  | 6 |
| Agus, D. B. | 2002 | + |  |  |  |  |  | + | + |  |  | + |  | 4 |
| Anido, J. | 2006 | + |  |  |  |  | + | + |  |  |  | + | + | 5 |
| Arnal-Estape, A. | 2010 | + | + |  |  | + |  | + | + |  |  | + |  | 6 |
| Barok, M. | 2008 | + |  |  |  |  |  |  | + |  |  | + |  | 3 |
| Barok, M. | 2007 | + |  |  |  |  |  | + | + |  |  | + |  | 4 |
| Baselga, J. | 1998 | + |  |  |  |  |  |  | + |  |  | + |  | 3 |
| Beyer, I. | 2011 | + |  |  |  | + |  | + | + |  |  |  | + | 5 |
| Bocangel, D. | 2006 | + |  |  |  |  |  |  | + |  |  | + | + | 4 |
| Brodie, A. | 2007 | + |  |  |  |  | + |  | + |  |  | + |  | 4 |
| Capietto, A. H. | 2011 | + | + |  |  | + |  | + | + |  |  | + | + | 7 |
| Chakrabarty, A. | 2013 | + | + |  |  | + |  |  | + |  |  | + | + | 6 |
| Chakrabarty, A. | 2012 | + |  |  |  | + | + |  | + |  | + | + | + | 7 |
| Chan, C. H. | 2012 | + |  |  |  |  |  | + | + |  |  | + | + | 5 |
| Lai, H. W. | 2012 | + | + |  |  | + |  |  | + |  |  |  | + | 5 |
| Cheung, N. K. | 2002 | + |  |  |  |  | + |  | + |  |  |  |  | 3 |
| Ching, C. L. | 2009 | + | + |  |  | + | + | + | + |  |  | + | + | 8 |
| Chiu, G. N. | 2007 | + |  |  |  |  |  |  | + |  |  |  | + | 3 |
| Chumsri, S. | 2011 | + |  |  |  |  |  | + |  |  |  |  | + | 3 |
| Colbern, G. T. | 1999 | + | + |  |  |  | + | + | + |  | + |  | + | 7 |
| Costantini, D. L. | 2010 | + |  |  |  |  | + | + | + |  |  |  | + | 5 |
| Damiano, V. | 2009 | + | + |  |  | + |  | + | + |  | + | + |  | 7 |
| Foy, K. C. | 2012 | + |  |  |  | + |  | + |  |  |  |  |  | 3 |
| Francia, G. | 2009 | + |  |  |  | + |  | + | + |  |  | + | + | 6 |
| Fujimoto-Ouchi, K. | 2002 | + | + |  |  |  | + | + | + |  |  | + | + | 7 |
| Fujimoto-Ouchi, K. | 2010 | + | + |  |  |  |  | + | + |  |  | + | + | 6 |
| Garrett, J. T. | 2012 | + | + |  |  | + |  | + | + |  | + | + | + | 8 |
| Gee, M. S. | 2008 | + |  |  |  | + | + | + | + |  |  | + | + | 7 |
| Gee, M. S. | 2007 | + |  |  |  |  | + | + | + |  |  | + | + | 6 |
| Gijsen, M. | 2010 | + | + |  |  | + |  |  | + |  |  | + | + | 6 |
| Han, H. | 2013 | + |  |  |  | + | + | + | + |  | + | + | + | 8 |
| Heyerdahl, H. | 2012 | + | + |  |  | + | + | + | + |  | + | + | + | 9 |
| Inoue, S. | 2011 | + |  |  |  | + |  | + | + |  |  | + | + | 6 |
| Ithimakin, S. | 2013 | + |  |  |  | + |  | + |  |  |  | + | + | 5 |
| Jerome, L. | 2006 | + | + |  |  |  |  |  | + |  |  | + | + | 5 |
| Jumbe, N. L. | 2010 | + | + |  |  |  | + | + | + |  | + | + | + | 8 |
| Junttila, T. T. | 2009 | + | + |  |  |  |  | + | + |  | + | + | + | 7 |
| Junttila, T. T. | 2011 | + | + |  |  | + |  | + | + |  |  |  | + | 6 |
| Junttila, T. T. | 2010 | + | + |  |  | + |  | + | + |  |  | + | + | 7 |
| Klos, K. S. | 2003 | + |  |  |  |  |  |  | + |  |  | + | + | 4 |
| Kodack, D. P. | 2012 | + |  |  |  | + |  | + | + |  |  | + | + | 6 |
| Kohrt, H. E. | 2012 | + | + |  |  | + |  | + | + |  |  | + | + | 7 |
| Kramer-Marek, G. | 2012 | + |  |  |  | + |  |  | + |  |  | + | + | 5 |
| Kute, T. E. | 2009 | + | + |  |  |  | + | + | + |  |  | + | + | 7 |
| Le, X. F. | 2008 | + |  |  |  |  |  |  | + |  |  | + | + | 4 |
| Lee, S. | 2002 | + |  |  |  |  |  |  | + |  |  |  | + | 3 |
| Lee-Hoeflich, S. T. | 2008 | + |  |  |  | + | + | + | + |  |  |  | + | 6 |
| Lewis Phillips, G. D. | 2008 | + | + |  |  | + | + | + | + |  | + | + | + | 9 |
| Liang, K. | 2010 | + |  |  |  |  | + | + | + |  |  | + | + | 6 |
| Liu, X. | 2006 | + |  |  |  |  | + |  | + |  |  | + | + | 5 |
| Lu, C. H. | 2007 | + |  |  |  |  |  |  | + |  |  |  | + | 3 |
| Magnifico, A. | 2009 | + |  |  |  | + |  | + | + |  |  | + | + | 6 |
| Mason, J. K. | 2013 | + |  |  |  |  | + | + | + |  |  | + | + | 6 |
| McKenzie, T. | 2004 | + |  |  |  |  |  |  | + |  |  | + | + | 4 |
| McLarty, K. | 2009 | + | + |  |  |  | + | + | + |  |  | + | + | 7 |
| Miller, T. W. | 2009 | + | + |  |  | + |  | + | + |  |  | + | + | 7 |
| Moulder, S. L. | 2001 | + | + |  |  |  |  |  | + |  |  | + | + | 5 |
| O'Donovan, N. | 2011 | + | + |  |  |  | + |  | + |  |  | + | + | 6 |
| Oliveras-Ferraros, C. | 2012 | + | + |  |  | + |  | + | + |  |  | + | + | 7 |
| Ono, N. | 2012 | + | + |  |  | + |  | + | + |  |  | + | + | 7 |
| Rasaneh, S. | 2012 | + | + |  |  |  |  |  | + |  | + |  | + | 5 |
| Reyzer, M. L. | 2004 | + | + |  |  |  | + | + | + |  |  | + | + | 7 |
| Ritter, C. A. | 2004 | + |  |  |  |  |  |  | + | + |  |  | + | 4 |
| Ritter, C. A. | 2007 | + | + |  |  |  |  |  | + |  |  | + | + | 5 |
| Rodrigues, L. M. | 2004 | + |  |  |  |  | + |  |  |  |  | + | + | 4 |
| Sabnis, G. | 2009 | + |  |  |  | + | + | + | + |  |  | + | + | 7 |
| Scaltriti, M. | 2007 | + |  |  |  |  | + | + | + |  |  | + | + | 6 |
| Scheuer, W. | 2009 | + |  |  |  | + |  | + | + |  |  | + | + | 6 |
| Scotti, M. L. | 2008 | + |  |  |  |  |  | + | + |  |  | + | + | 5 |
| Seoane, S. | 2010 | + | + |  |  | + |  |  | + |  |  | + | + | 6 |
| Shen, G. | 2011 | + |  |  |  | + |  | + | + | + | + | + | + | 8 |
| Smith, T. A. | 2013 | + |  |  |  | + | + |  | + |  |  | + | + | 6 |
| Spiridon, C. I. | 2002 | + |  |  |  |  |  |  | + | + |  | + |  | 4 |
| Wang, C. X. | 2005 | + |  |  |  |  |  |  | + |  |  | + | + | 4 |
| Wang, L. H. | 2007 | + | + |  |  |  |  | + | + |  |  | + | + | 6 |
| Wang, S. | 2012 | + | + |  |  | + |  | + | + |  |  | + | + | 7 |
| Warburton, C. | 2004 | + |  |  |  |  | + | + | + |  |  | + |  | 5 |
| Waterhouse, D. N. | 2005 | + |  |  |  |  | + |  | + | + |  | + | + | 6 |
| Wen, X. F. | 2006 | + | + |  |  |  | + |  | + |  |  | + | + | 6 |
| Zhang, N. | 2011 | + | + |  |  |  |  |  | + |  |  | + | + | 5 |
| Zhao, Y. | 2011 | + | + |  |  |  |  | + | + |  |  | + | + | 6 |
| Zhu, Y. | 2012 | + | + |  |  | + | + |  | + |  |  | + | + | 7 |
| Zhuang, G. | 2010 | + |  |  |  | + |  |  | + |  |  | + | + | 5 |
