# Supplementary material for: The Efficacy of Trastuzumab in Animal Models of Breast Cancer: A Systematic Review and Meta-Analysis
Source: PLoS One. 2016 Jul 27;11(7):e0158240. doi: 10.1371/journal.pone.0158240 (PMC4963137; doi:10.1371/journal.pone.0158240)
Supplement: S1 Text — (PDF) [file pone.0158240.s007.pdf]

# A systematic review and meta-analysis of trastuzumab in the treatment of animal models of breast cancer

Jiarong Chen[1], Canhong Yang[2], Bin Guo[3], Emily Sena[4], Malcolm Macleod[4], Yawei Yuan[1], Theodore Hirst[4]

*1: Department of Radiation Oncology, Nanfang Hospital, Southern Medical University, 1838 Guangzhou DaDao Bei, Guangzhou, Guangdong 510515, P.R. China*
*2: Department of Neurology, the Third Affiliated Hospital of Southern Medical University, Guangzhou 510630, P.R. China*
*3: Department of Hepatobiliary Surgery, Zhujiang Hospital, Southern Medical University, Guangzhou 510282, P.R. China*
*4: Centre for Clinical Brain Sciences, Chancellors Building, University of Edinburgh, 49 Little France Crescent, Edinburgh EH16 4SB, UK*

**Introduction:**

Breast cancer is the most common malignancy in women worldwide. Trastuzumab has established efficacy against breast cancer with overexpression or amplification of the HER2 oncogene by RCTs. However, no analysis has concluded the efficiency of trastuzumab on animal studies and whether this data is confounded by limitations in study quality.

**Objectives:**

We aim to quantify the therapeutic effect of trastuzumab in animal research and the relationship of this to the HER2 status of the tumour treated. We aim to provide an estimate of trastuzumab efficacy and the impact of study design features by including only high quality studies at low risk of bias.

**Hypothesis:**

We hypothesize that trastuzumab is effective in animal studies across a range of study design variables, that a large proportion of studies are of poor quality i.e. do not randomize/blind and that publication bias is evident from the dataset.

**Search strategy:**

We will search PubMed, Medline and EMBASE. Studies in all languages accepted. Search terms: (breast tumor OR breast cancer) AND (trastuzumab OR Herceptin)

*In October 2014 we added two extra terms: (breast tumor OR breast cancer) AND (trastuzumab OR Herceptin OR rhuMAb HER2" OR "Anti-p185HER2 Monoclonal Antibody" OR "muMAb 4D5"). The added terms were previous names for trastuzumab. This search returned 16 studies and resulted in the inclusion of 1 study.*

**Inclusion criteria:**

We will include controlled studies that report the monotherapy efficacy of trastuzumab in experimental animals with breast cancer model utilized. Outcome should report as either median survival or tumor volume with the mean effect size and its variance. The number of animals per group should be stated within the publication. All “Reviews”, “Books”, “Letters”, “Clinical trials”, “Case reports”, or “Editorials” were excluded.

**Quality assessment:**

Two review authors will independently assess the risk of bias in included studies by considering the following characteristics: randomized allocation of tumour-bearing animal into treatment and control groups, blinded assessment of outcome, sample size calculation performed, compliance with animal welfare policy, explanation of rationale for disease model used, or more than one model assessed for comparison, standardised number or volume of tumour cells implanted, reported number of animals in which the tumour did not grow, reporting and explanation of excluded animals, presentation of evidence that trastuzumab acts directly against the tumour, consistent implantation site

**Data extraction:**

Titles and/or abstracts of studies retrieved using the search strategy and those from additional sources will be screened independently by two review authors to identify studies that potentially meet the inclusion criteria outlined above. The full text of these potentially eligible studies will be retrieved and independently assessed for eligibility by two review team members (Canhong Yang and Bin Guo). Any disagreement between them over the eligibility of particular studies will be resolved through discussion with a third reviewer (Jiarong Chen) after full text reading.

The CAMARADES data manager will be used to extract data from the included

studies for assessment of study quality and evidence synthesis. Extracted information will include: article information (title, author, journal, publication year); agent for therapeutic and control group; trastuzumab treatment (dose, total dose and frequency, route of administration, delay to treatment); tumor volume data for therapeutic and control group (mean, s.d. or s.e. (Missing data will be requested from study authors.), original tumor size included, measurement method and frequency, measurement day after treatment, measurement day after tumour implantation,blinded assessment of outcome ); overall survival data for therapeutic and control group (mean, s.d. or s.e.); experimental animals(number of animals for every group, species and strain, age, sex and original weight), breast cancer model (breast cancer cell type, tumour implantation method and implantation site, number or volume of implanted tumour cells)

**Analysis:**

1) Volume data:

We will log-transform raw mean values and generate log transformed SEs using the formula (log(mean+1.96(SE))-log(mean-1.96(SE)))/(2*1.96). This is to account for the exponential nature of tumour growth. Following this we will generate a percentage effect score for each experiment using the formula 100*(control-treatment)/control. We will then collate generate a global efficacy estimate using DerSimonian and Laird meta-analysis and use stratified meta-analysis to identify sources of between-study heterogeneity

*Through late 2014-early 2015, we had several discussions about whether or not to log-transform the normalized mean difference statistic. Ultimately we agreed that applying the normalized mean difference formula* 100*(control-treatment)/control *would produce nonparametric data and, with a large number of studies reporting efficacies with NMD statistics greater than +90%, there would be a resultant clustering of studies close to the ceiling efficacy of +100%. Therefore we decided to log transform the dataset to produce a parametrically distributed ratio statistic without ceiling. Ultimately we elected to adopt an alternative summary statistic – based on log-transformed data – as suggested by Higgins et al(Higgins et al., 2008), as this would provide more intuitive output data.*

*We will log-transform mean volumes using the following formula:*

$$\bar{z}_i = \ln(\bar{x}_i) - \frac{1}{2}\ln\left(\frac{s_{x,i}^2}{x_i^2} + 1\right) \quad \textit{(i=Rx,C)}$$

*and calculate summary statistic for efficacy:*

$$ES_z = \bar{z}_{Rx} - \bar{z}_C$$

*We will log-transformed standard deviations:*

$$s_{z,i} = \sqrt{\ln\left(\frac{s_{x,i}^2}{\bar{x}_i^2} + 1\right)} \quad \textit{(i=Rx,C)}$$

*and calculate variance as follows:*

$$var(\bar{z}_i) = \frac{s_{z,i}^2}{n_i} \quad \textit{(i=Rx,C)}$$

*Finally we will generate study standard error as following:*

$$SE(ES_z) = \sqrt{var(\bar{z}_{Rx}) + var(\bar{z}_C)}$$

*We will then use DerSimonian and Laird random effects meta-analysis to pool studies and stratified meta-analysis to search for sources of heterogeneity. For each group we will delog output values to give intuitive values for mean and SE.*

2) Survival data

We will generate an effect score of median survival ratio by dividing the treated group by control group. We will then log-transform the data and use a modified form of DerSimonian and Laird meta-analysis (weighted by the number of animals in the study instead of inverse variance) to generate a global efficacy estimate. We will used stratified meta-analysis as above

Publication bias: We will use funnel plots, Egger regression and Trim and Fill analysis to search for evidence of publication bias.

## References

HIGGINS, J. P., WHITE, I. R. & ANZURES-CABRERA, J. 2008. Meta-analysis of skewed data: combining results reported on log-transformed or raw scales. *Stat Med,* 27**,** 6072-92.
